# Genome-wide association study reveals significant genomic regions for improving yield, adaptability of rice under dry direct seeded cultivation condition

**DOI:** 10.1186/s12864-019-5840-9

**Published:** 2019-06-10

**Authors:** Sushil Raj Subedi, Nitika Sandhu, Vikas Kumar Singh, Pallavi Sinha, Santosh Kumar, S. P. Singh, Surya Kant Ghimire, Madhav Pandey, Ram Baran Yadaw, Rajeev K. Varshney, Arvind Kumar

**Affiliations:** 1 Rice Breeding Platform, International Rice Research Institute, DAPO Box 7777, Metro Manila, Philippines; 2 International Rice Research Institute, South Asia Hub, ICRISAT, Patancheru, Hyderabad, India; 3 Center of Excellence in Genomics and System Biology, International Crops Research Institute for the Semi-Arid Tropics (ICRISAT), Patancheru, Hyderabad, India; 4 ICAR Research Complex for Eastern Region, Patna, Bihar India; 5 Bihar Agricultural University, Sabour, Bihar India; 6 Agriculture and Forestry University, Rampur, Chitwan Nepal; 7 National Rice Research Program, Hardinath, Nepal; 8 Punjab Agricultural University, Ludhiana, India

**Keywords:** Dry direct seeded, Marker-trait association, Nutrient uptake, Rice, Root, Yield

## Abstract

**Background:**

Puddled transplanted system of rice cultivation despite having several benefits, is a highly labor, water and energy intensive system. In the face of changing climatic conditions, a successful transition from puddled to dry direct seeded rice (DDSR) cultivation system looks must in future. Genome-wide association study was performed for traits including, roots and nutrient uptake (14 traits), plant-morphological (5 traits), lodging-resistance (4 traits) and yield and yield attributing traits (7 traits) with the aim to identify significant marker-trait associations (MTAs) for traits enhancing rice adaptability to dry direct-seeded rice (DDSR) system.

**Results:**

Study identified a total of 37 highly significant MTAs for 20 traits. The false discovery rate (FDR) ranged from 0.264 to 3.69 × 10^− 4^, 0.0330 to 1.25 × 10^− 4^, and 0.0534 to 4.60 × 10^− 6^ in 2015WS, 2016DS and combined analysis, respectively. The percent phenotypic variance (PV) explained by SNPs ranged from 9 to 92%. Among the identified significant MTAs, 15 MTAs associated with the traits including nodal root, root hair length, root length density, stem and culm diameter, plant height and grain yield were reported to be located in the proximity of earlier identified candidate gene. The significant positive correlation of grain-yield with seedling establishment traits, root morphological and nutrient-uptake related traits and grain yield attributing traits pointing towards combining target traits to increase rice yield and adaptability under DDSR. Seven promising progenies with better root morphology, nutrient-uptake and higher grain yield were identified that can further be used in genomics assisted breeding for DDSR varietal development.

**Conclusions:**

Once validated, the identified MTAs and the SNPs linked with trait of interest could be of direct use in genomic assisted breeding (GAB) to improve grain yield and adaptability of rice under DDSR.

**Electronic supplementary material:**

The online version of this article (10.1186/s12864-019-5840-9) contains supplementary material, which is available to authorized users.

## Background

Developing resource-water-labor-energy efficient, dry direct seeded adapted rice varieties under reducing water-labor availability in agriculture is necessary for the sustainability of world’s future food security [1]. The continuous efforts involving yield improvement under direct seeded conditions through better exploitation of genetic and genomic resources may lead to a breakthrough in improving rice yield and adaptability under dry direct seeded rice (DDSR) situation. The rapid development of next-generation sequencing (NGS) technologies has opened new avenues to harness existing genetic diversity to better understand the genetic basis of targeted traits and to deploy in genomics-assisted breeding (GAB) program [2, 3]. Advanced genotyping technologies offer opportunities to plant breeding community to sequence every individual of GAB in several folds of crop genome at lower cost [4].

Water-saving technologies such as SRI (system of rice intensification) [5], AWD (alternate wetting and drying) [6] and DDSR (dry direct seeded rice) [7] can be advocated as suitable alternative to PTR. DDSR has the potential to ensure overall sustainability of rice cultivation systems when applied as a full package comprising laser-assisted land levelling, conservation tillage, crop establishment, harvesting and processing using smart machines, integrated weed management, precision input delivery especially of water and nutrients and suitable varieties. DDSR accounted for 28% area in India, 45% in Korea, 39 to 47% in Vietnam and more than 90% collectively in United States, Sri Lanka, and Malaysia [8]. The mechanized DDSR method of rice cultivation has been estimated to provide a potential irrigation water savings of 40 cm ha^− 1^, labor savings of 25 person-days ha^− 1^, energy savings of 1500 MJ ha^− 1^, a reduction of GHG emissions of 1500 kg CO_2_ equivalent ha^− 1^, yield increase of 0.5 t ha^− 1^ and increased net economic return of USD 50 ha^− 1^ with positive effect on soil heath in most of the rice-growing countries [8]. DDSR combines the advantage of better adaptation of upland rice varieties in aerobic soil and high yield potential of lowland varieties adapted to anaerobic soil.

A number of plant morphological features such as leaf size, thickness, angle, shape [9] play a remarkable role in photosynthesis [10], flowering time [11–13], lodging resistance [14] and plant height [15] and ultimately determine the crop productivity. The phenotypic root plasticity, root morphology and plant architecture largely affect crop productivity and therefore represent the key targets for the marker-assisted breeding schemes designed for increasing yield, biotic/abiotic stress tolerance and grain quality [16–18]. DDSR faces the key challenge of reduced nutrient uptake especially nitrogen, phosphorus and iron [19] than traditional puddled rice cultivation system. The lower uptake of nutrients create adverse effect on plant agronomic traits such as height, growth rate, tillering ability, leaf area index, spikelet number, grain filling and physiological processes such as photosynthesis, respiration and hormonal metabolism [20]. Lodging has been found as one of the major constraints in attaining high yield in DDSR compared to puddled system of rice cultivation. Moderate plant height, culm and stem diameter, thickness, length and strength are important traits contributing to lodging resistance under DDSR [21]. Root development and root system architecture is highly responsive to nutrient availability [18, 19, 22] and root traits reported to be associated with water uptake and grain yield in rice [23, 24], wheat [25], maize [26] and soybean [27]. Addressing the challenge of appropriate plant and root architecture, efficient nutrient uptake, early vegetative vigor, early and uniform emergence, lodging resistance under DDSR and dissecting the genetic basis to maximize the potential to breed high-yielding resource-efficient DDSR varieties using modern biotechnological and bioinformatic approaches is mandatory. A better knowledge of rice root system including number of nodal roots, lateral roots, root length density, root biomass and root branching with more root hairs is must. The proper understanding of the relationship of these root morphological traits with nutrient uptake and grain yield may be important to the development of rice varieties with flexibility to be grown under variable situations ranging from wet and dry direct seeded to transplanted situations.

Genotyping by sequencing (GBS) has allowed breeders and researchers to track chromosome segments and marker-trait associations in QTL-mapping and genome-wide association studies (GWAS) [28]. The emergence of cheap, high-throughput genotyping platforms like GBS, that have sufficient marker density can be used successfully in association mapping studies in complex crop species including wheat [29], foxtail millet [30], soybean [31], maize [32, 33], rice [34], chickpea [35] and pigeonpea [36, 37].

Genome wide association mapping has been reported as an effective method to fine map the complex traits contributing to productivity in crop species [34, 38]. GWAS can be applied to study the statistical marker-trait associations involving diverse germplasm, landraces, multi-parent breeding population and NAM (nested association mapping population) populations [39]. Identification of cost-effective, easy to use, widely distributed, co-dominant, phenotype-associated and regulatory SNPs, candidate genes and regulatory pathways could represent a significant milestone to accelerate the global hunt to improve rice yield under DDSR. Crop improvement programs can use association studies to access the allelic diversity and to identify the best alleles to be assembled in developing superior DDSR adapted rice varieties. A well-defined image of traits needed for DDSR, linked SNPs and functional characterization of underlying candidate genes will help rice breeders to choose specific donors, recipients and traits to undertake a systematic breeding program enhancing adaptability and improving rice yield under DDSR.

In the present study, we performed GWAS for 39 traits including seedling establishment traits, root and plant morphological traits, yield and yield attributing traits in a MAGIC (Multi-parent advanced generation inter-cross) population with the aim to identify significant marker-trait associations to be used directly to breed high yielding and nutrient-efficient rice varieties for DDSR condition.

## Methods

### Development of MAGIC population

**Fig. 1:**
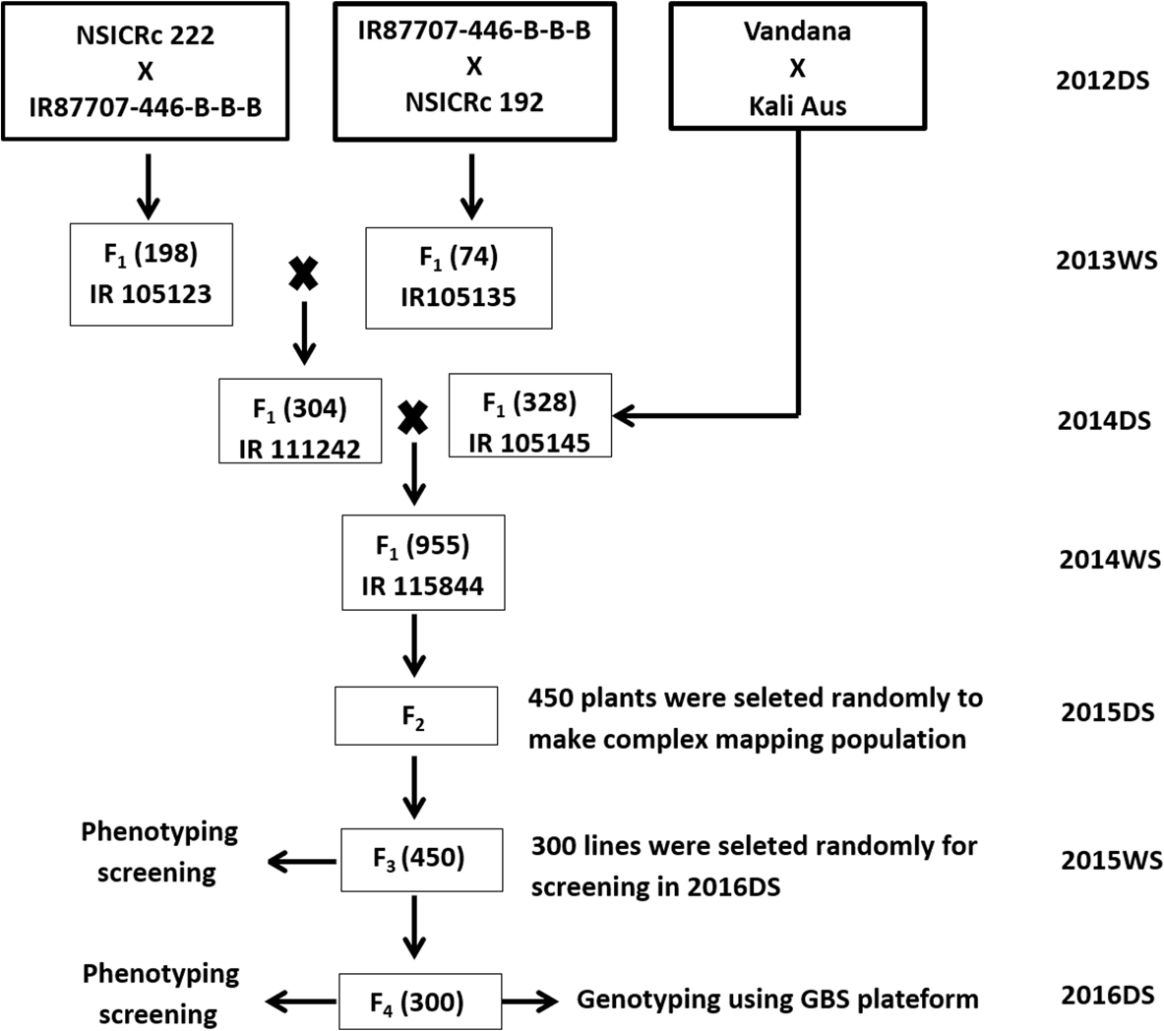
The breeding scheme for the development of MAGIC population and number of plants selected at each stage for the phenotyping under the direct seeded condition and for genotyping using GBS platform

The MAGIC population used in the study was developed by the inter-crossing of five diverse parents: IR 87707–446-B-B-B, NSICRc 222, IR 74371–54–1-1, Vandana and Kali Aus that has been characterised for upland adaptability (NSICRc 192, Vandana, Kali Aus), better root system (Kali Aus), drought tolerance (IR 87707–446-B-B-B, NSICRc 192, Kali Aus) and high yield under lowland adapted rainfed environment (NSICRc 222). The detailed description on trait characterstictics of parental progenies is presented in Table 1. These parents were intermated and recombined through further selfing to develop a MAGIC population comprised of 300 progenies. The detailed schematic representation of the development of the MAGIC population and phenotyping and genotyping screening is provided in Fig. 1.

**Table 1:** Description of the parental lines used for developing the MAGIC population

S.N.	Parental Line	Country of origin	Agronomic Relevance
1	NSICRc 222	Philippines	High yielding irrigated variety (5.0–8.0 t ha^− 1^), 105 to 110 days to maturity
2	IR 87707–446-B-B-B	Philippines	Drought tolerant rice variety in IR64 background released in Nepal as Sukha dhan 4 in 2014 (125 days to maturity) and Myanmar as Yaenelo 4 in 2015 (115 days to maturity) and an average grain yield of 4.0 to 5.0 t ha^− 1^
3	NSICRc 192	Philippines	High yielding drought tolerant rice varieties (3.7–5.5 t ha^− 1^) released as Sahod Ulan 1 in the Philippines in 2009 (110 days to maturity)
4	Vandana	India	Semi-tall, white kernels with long and bold grain, upland adapted rice variety, and an average grain yield of 4.0 to 5.0 t ha^− 1^
5	Kali Aus	India	Medium-duration drought tolerant, long and deep root system and an average grain yield of 4.0 to 5.0 t ha^− 1^

### Field experiments

The field experiments were conducted in upland farm fields at the International Rice Research Institute (IRRI), Los Banos, Laguna, Philippines (14^0^ 10^′^11.81^″^N, 121^0^ 15^′^ 22^″^ E) in 2015 wet season (2015WS) and 2016 dry season (2016DS). The data on average rainfall, temperature, humidity, solar radition and pressure across 2015WS and 2016DS is presented in Additional file 2: Figure S1. In the present study, the term “dry direct-seeded” refers to the sowing of rice directly in the dry field without any nursery-bed raising in non-puddled, well-levelled fields. To ensure better pulverization, better germination and weed control, the field was prepared 1 month prior to sowing. Furrow was prepared using a tractor, and basal fertilizer was incorporated in the soil. To control weeds, combination of pre-emergence (Oxadiazon @ 0.5 kg ai ha^− 1^ at 6 days after seeding (DAS)), early post emergence (Bispyribac sodium @ 0.03 kg ai ha^− 1^ (9.7%, nominee) at 11 and 22 DAS) followed by spot weeding at 35 and 55 DAS was used. Integrated pest management (IPM) practices involving rat baiting using ditrac @ 0.05 g kg^− 1^ = 0.005% brodifacoum bait to control rats with the pre-seeding application of Fipronil @ 0.075 kg ai ha^− 1^ along the bunds and at 7 DAS along the plot edges was followed. The seeding was done at ~2 cm depth in furrows in the dry plots. The seeding density was 2 g per progeny (approximately 30–35 seeds per row) for the two rows. Out of total 3 m row, the central 2 m was used for the measurement of grain yield and yield related traits and remaining 1 m (0.5 m at both side) was used for destructive sampling for the measurement of root traits at different growth stages. Sprinkler method of irrigation was used during the seedling establishment stage (1 to 21 DAS) and thereafter surface irrigation was applied once or twice a week depending on the weather and crop water status. Then irrigated field was allowed to drain naturally through normal seepage and percolation.

#### 2015WS

A total of 450 F_2_ derived F_3_ progenies with significant phenotypic variability for grain yield and yield attributing traits along with five parents and five checks were evaluated in an augmented design (100 progenies per block × 5 blocks) under DDSR conditions. The total fertilizer rate was 100-35-30 NPK kg ha^− 1^.

#### 2016DS

A total of 300 F_3_ derived F_4_ progenies with significant phenotypic variability for grain yield and yield attributing traits along with five parents and five checks were evaluated in an alpha-lattice design (5 progenies per block × 62 blocks) with two replications under DDSR conditions. The total fertilizer rate was 120-40-40 NPK kg ha^− 1^.

The detailed description of experiments conducted and traits measured under the present study is presented in Table 2.

**Table 2:** Detail of the experiments conducted and traits measured during 2015WS and 2016DS under dry direct seeded conditions

SN	Year/ Season	Date of seeding	No. of Entries	Parents and checks^a^	Observed traits
1	2015WS	June 18, 2015	500 (450 + 50)	NSICRc 222, IR 87707–446-B-B, IR 74371–54–1-1, Vandana/Kali Aus, IR 94225-B-82-B^a^ IR 94226-B-177-B^a^ IR 91648-B-32-B^a^ IR 91648-B-153-B^a^ IR 91648-B-230-B^a^	days to first emergence, days to full emergence, number of nodal roots at 15 DAS, number of nodal roots at 22 DAS, number of nodal roots at 29 DAS, maximum root length (cm) at 15 DAS, maximum root length (cm) at 22 DAS, maximum root length (cm) at 29 DAS, dry shoot weight at 15 DAS (g), dry shoot weight at 22 DAS (g), dry shoot weight at 29 DAS (g), relative growth rate from 15 to 22 DAS, relative growth rate from 22 to 29 DAS, relative growth rate from 15 to 29 DAS, root hair length, root hair density, flag leaf length, flag leaf width, flag leag area, Flag leaf angle, leaf color chart, cholorophyll content (SPAD), stem diameter, culm diameter, bending strength (kg cm), bending moment (kg cm^2^), plant height (cm), days to 50% flowering, biomass at 50% flowering (g), vegetative vigor score, number of productive tillers per plant, panicle length (cm), number of filled grains/panicle, 1000 grain weight (g), straw yield (kg ha^− 1^), grain yield (kg ha^− 1^), N uptake (kg ha^− 1^), P uptake (kg ha^− 1^), Fe uptake (kg ha^− 1^), Zn uptake (kg ha^− 1^)
2	2016DS	Dec 22, 2015	310 (300 + 10)		days to first emergence, days to full emergence, number of nodal roots at 15 DAS, number of nodal roots at 22 DAS, number of nodal roots at 29 DAS, maximum root length (cm) at 15 DAS, maximum root length (cm) at 22 DAS, maximum root length (cm) at 29 DAS, dry shoot weight at 15 DAS (g), dry shoot weight at 22 DAS (g), dry shoot weight at 29 DAS (g), relative growth rate from 15 to 22 DAS, relative growth rate from 22 to 29 DAS, relative growth rate from 15 to 29 DAS, root hair length, root hair density, flag leaf length, flag leaf width, flag leag area, Flag leaf Angle, leaf color chart, cholorophyll content (SPAD), stem diameter, culm diameter, bending strength (kg cm), bending moment (kg cm^2^), plant height (cm), days to 50% flowering, biomass at 50% flowering (g), vegetative vigor score, number of productive tillers per plant, panicle length (cm), number of filled grains/panicle, 1000 grain weight (g), straw yield (kg ha^− 1^), grain yield (kg ha^− 1^)

*WS* wet season, *DS* Dry season, *DAS* days after seeding (DAS)

IR 94225-B-82-B (Aus276/3*IR64 derived progenies with better root traits and grain yield under dry direct seeding conditions [19]; IR 94226-B-177-B (Aus276/3*MTU1010 derived progenies with better root traits and grain yield under dry direct seeding conditions [19]; IR 91648-B-32-B (Moroberekan/3* Swarna derived progenies with genetic region for early and uniform germination characteristics [40]; IR 91648-B-153-B and IR 91648-B-230-B (Moroberekan/3* Swarna derived progenies with genetic region for lodging resistance [40]

### Measurements of phenotypic traits

Five parental progenies, five checks and 300 progenies from the MAGIC population were characterized for a total of 39 agronomically important traits (Table 2; Additional file 2: Figure S2). These traits include the seedling establishment traits (9), root morphological and nutrient uptake traits (14), lodging resistance traits (4), plant morphological traits (5), grain yield and yield-related traits (7). Evaluation and characterization was done under DDSR in 2015WS and 2016DS at IRRI, Philippines.

#### Seedling establishment traits

Seedling emergence was recorded regularly from 3 to 12 days after seeding (DAS). Days to first emergence (DTFirst) refers to the emergence of seedling (one or two) from the soil and days to full emergence (DTFull) refers to the emergence of 90–95% of seedling per plot.

For early vegetative vigor, the randomly three seedlings per plot were uprooted from the soil using trowel at 15, 22 and 30 days after seeding. The roots and shoots were separated and the shoot was oven dried at 60 °C for 72 h. The oven dried shoot was then weighed to calculate RGR (Relative Growth Rate). RGR for 15 to 22 DAS (RGR1), 22 to 29 DAS (RGR2) and 15 to 29 DAS (RGR3) was calculated by using following formula [19].

$$ \frac{\log\ \left(\mathrm{dry}\ \mathrm{shoot}\ \mathrm{weight}\ \mathrm{at}\ \mathrm{sampling}\ 2\right)\hbox{-} \log \left(\mathrm{dry}\ \mathrm{shoot}\ \mathrm{weight}\ \mathrm{at}\ \mathrm{sampling}\ 1\right)}{\left(\mathrm{date}\ \mathrm{of}\ \mathrm{sampling}\ 2\hbox{-} \mathrm{dateof}\ \mathrm{sampling}\ 1\right)} $$

The separated roots were used for the root traits measurements. Vegetative vigor (VVG) was also recorded following 1–9 scale (1: extra vigorous, 3: vigorous, 5: normal, 7: weak, 9: very weak) at 30 DAS according to the IRRI Standard Evaluation System for Rice [41].

#### Measurements of root traits

For the measurement of root traits, the random three plants were removed from the soil at each sampling. The soil sections surrounding the plant containing roots were removed by digging a hole (40 cm deep). The roots and shoots were then separated by cutting from the top soil line. The separated roots were put into a sieve and washed gently with tap water. The number of Nodal roots (NR) [number of nodal roots at 15 DAS (NR1), number of nodal roots at 22 DAS (NR2), number of nodal roots at 29 DAS (NR3)] and maximum root length (RL) [maximum root length at 15 DAS (RL1), maximum root length at 22 DAS (RL2), maximum root length at 29 DAS (RL3)] were recorded. The nodal roots were counted manually, and the maximum root length was measured using ruler measured in centimetre. The measurements on root hair length and density were recorded on six root samples following the procedure as described by Sandhu et al. [19].

At booting stage, the Leaf Color Chart (LCC) developed by IRRI was used along with the chlorophyll meter (SPAD) (Minolta 502) to estimate and validate the relative accuracy of LCC in measuring greenness of fully expanded flag leaf nitrogen (N) concentration. The color of the flag leaf from the three randomly chosen plants was measured by placing the middle part of the middle lobe of the flag leaf in front of the color strip (LCC) for the comparison. The three phenotypically similar plants with approximately same value of LCC and SPAD were then uprooted for the total above-ground biomass measurement. The above ground fresh biomass was recorded immediately after uprooting, and then all the three plant samples were cut into small pieces and mixed properly, and a total of 200 g plant sample were taken for each progeny and then oven dried in 70 °C for 3 days and then weight for dry plant biomass and nutrient uptake measurements [19]. In 2015WS a total of 60 progenies (30 high yielding and 30 low grain yielding along with the parents) were selected and analyzed for nutrient (N, P, Fe and Zn) uptake at IRRI Analytical Service Laboratory. The nitrogen estimation was carried out using Kjeldahl digestion method. The plant material was digested with a mixture of K_2_SO_4_ (potassium sulphate) and concentrated H_2_SO_4_ (sulphuric acid) in presence of catalyst (fine powdered selenium). The NH_3_ (ammonia) produced was estimated by colorimetric method using Technicon autoanalyzer. The other nutrients phosphorus (P), Iron (Fe) and Zinc (Zn) were estimated using Nitric/Perchloric Acid digestion method using AIM 500 Digestion Block System.

#### Lodging resistance traits

Randomly three plants per plot were chosen for the lodging resistance traits measurements. Traits related to lodging resistance including stem diameter (SD; mm), culm diameter (CD; mm), bending strength (BS; kg cm) and bending moment (BM, kg cm^− 2^) were recorded. Stem and culm diameter were measured using vernier caliper. Stem strength at the breaking point was measured using the prostrate tester (Daiki Rika Kogyou Co., Tokyo; Additional file 2: Figure S3) [42]. At 20 days after flowering, the main stem of the plant was cut from the ground level and the force was given to the 2^nd^ and 3^rd^ internode of the stem at the middle (5 cm) of the internode. The dispacement in mm was measured as the stem strength at breaking point. The bending ability of internode was measured by bending the stem to point at which the stem break and the scale displacement (mm) due to bending ability was recorded.

#### Plant morphological, grain yield and yield-related traits

Five random plants were taken for recording of plant morphological, grain yield and yield-related traits. Days to 50% flowering (DTF) was recorded when ~ 50% of the plants in a plot showed panicle excertion. Plant height (PHT), panicle length (PL), flag leaf length (FLL) and flag leaf width (FLW) were measured using ruler in centimeter scale. The number of productive tillers per plant (NPT) were counted manually. The PHT was measured from the ground level to the tip of the highest panicle at the maturity stage. PL was measured from the node of the panicle to the tip of the panicle. The FLL and FLW was measured from the base to tip of flag leaf and at the middle part of the flag leaf, respectively. Flag leaf area (FLA; cm^2^) was calculated according to Palamiswamy and Gomez [43]. Flag leaf angle (FLAngle) was measured using protector keeping stem as a horizontal base. The harvested grain was threshed, cleaned and oven dried for 3 days at 50 °C to 14% moisture content and then the weighing to record grain yield. The filled grains per panicle (FG/P) were counted manually and the 1000 grain weight (1000GW) was recorded in g. For straw yield (SY), the harvested straw was oven dried for 3 days at 70 °C and weighed. The biomass at flowering (BMF) was measured (in g) after harvesting and drying the above ground biomass at 70 °C in oven till there is no change in the dry weight of the plants.

### DNA isolation, genotyping-by-sequencing and SNP calling

Samples comprised of the five parents, five checks and 300 progenies from the MAGIC population. The fresh leaf tissue samples from six plants per progeny were collected at 40 DAS. To increase the throughput and efficiency coupled with less chances of error, automated leaf sampling and high-throughput DNA extraction using the Brooks’ PlantTrak Hx rice leaf tissue sampler and LGC Genomics’ oKtopure systems was used. For genotyping by sequencing (GBS) a type II restriction endonuclease *ApeKI* was used for DNA digestion, and the digested DNAs were ligated to the adapter, and then 96-plex library was constructed as per GBS protocol. GBS was carried out using HiSeq2000 100PE platform of Macrogen Inc. (Korea). From the GBS data a total of 2,75,586 SNPs were called from the GBS genotyping. A stringent selection criterion to filter out the SNPs was used including missing percentage, MAF (minor allele frequency) and percent heterozygosity to select the panel of robust SNPs. SNP with call rate 80% and MAF (minor allele frequency) of > 5% (24,306 SNPs) were filtered using Tassel 5. The 24,306 SNPs were used to estimate the genetic relationship, the building of neighbor Joining tree and were used for GWAS for emergence, root, grain yield and yield-related traits. To detect and correct for population structure, a PCA was carried out using 24,306 SNP markers.

### Phenotypic data analysis

Analysis of variance (ANOVA) was computed using PBTools V 1.4.0 (http://bbi.irri.org/products). The trial mean and trait mean for each season/years was computed using mixed model analysis considering replications, and block within replication as random effect and progenies as a fixed effect. The broad-sense heritability was computed using the equation, H = σ^2^_G_/(σ^2^_G_ + σ^2^_E_/r).

Where H represents the broad sense heritability, σ^2^G represents the genetic variance, σ^2^E the error variance and r the number of replications. Correlation among traits was calculated using function rcorr() [in Hmisc package] in R. v.1.1.423.

The best parent for the promising traits was selected and the percent improvement in selected promising breeding progenies over the best parent for each promising trait was calculated as: % improvement = (trait value in selected promising progeny – trait value in best parent/trait value in best parent) *100.

### Population structure, linkage disequilibrium and association analysis

The 300 progenies and five parents included in the present study include the progenies of *indica* and *Aus* parents. The population structure was estimated using STRUCTURE V.2.3.4. Using 24,306 SNPs, a series of models with *K* value ranging from 1 to 10 was run with a burn-in period to 50,000 and running length to 10,000 to give consistent results overruns. To verify the consistency and accuracy of the results, ten independent runs for each *K* was performed. The *K* value with maximum likelihood over the runs was considered as the most probable number of clusters [44]. The genetic structure of the population was also described on Principal components (PC) calculated with R/GAPIT [45] and iteratively added to fixed part of the model, ranging from PC1 to PC10. The distance matrix was calculated using Tassel 5.0 [46], and an unweighted neighbor-joining tree was visualized using FigTree v1.4.2 [47]. The pairwise r^2^ values were estimated using Tassel 5.0 and were averaged over the SNPs grouped based on stepwise increasing base-pair distance (0–10, 10–50, 50–500, 500–1000, 1000–1500, 1500–2000, 2000–2500, 2500–3000, 3000–3500, 3500–4000, 4000–4500 and 4500–5000). The average LD over these base-pair distances was used to estimate the linkage disequilibrium (LD) decay in the MAGIC population. GAPIT (Genome Association and Prediction Integrated Tool) was used to conduct genome wide association studies on 2015WS, 2016DS and the combined data over seasons to identify the genomic regions associated with the traits of interest [45].

The allelic effects of the significantly associated markers for root, nutrient uptake, and grain yield traits were determined by representing phenotypic data for the alleles as boxplots and the significant allelic variation for the associated traits was determined to perform Kruskal–Wallis test in “R**.”**

### Correction of false discovery rate (FDR) and candidate gene prediction

To correct the false positive in genome wide association analysis even keeping the stringent *p*-value benchmark, “Bonferroni Correction” the most stringent correction method was used. After the Bonferroni multiple test correction (0.01/24,306; significance level of 1%/total number of marker used in analysis), the calculated threshold value was 4.1 × 10^− 7^. Only the marker-trait associations (MTA) that surpass the Bonferroni threshold and consistent over seasons for the recorded traits were reported. For the identified significant marker-trait associations, candidate gene prediction was performed within 120-kb region around i.e. 120 kb upstream and 120 kb downstream of the SNPs with significant association signals. Earlier reported QTL that co-localized with the present study SNPs/QTLs, literature searches were performed. Regions covered by SNPs were searched for candidate genes using the MSU v.7 rice genome browser (http://rice.plantbiology.msu.edu/cgi-bin/gbrowse/rice/#search) and QTARO database (http://qtaro.abr.affrc.go.jp).

## Results and discussion

### Phenotypic variations for targeted raits

A number of seedling establishment, root and leaf morphological, agronomic, grain yield and yield related traits were measured to investigate their role in improving rice adaptation under DDSR condition. A better understanding of root traits enhancing nutrient uptake under DDSR in the framework of breeding strategies is a significant step forward to increase nutrient uptake under DDSR condition. Significant variations were observed for all the selected traits in both the seasons, except for number of filled grains per panicle, number of productive tillers per plant, panicle length and total biomass at 50% flowering (Table 3). The mean grain yield of the population was 3769 kg ha^− 1^ in 2015WS and 4472 kg ha^− 1^ in 2016DS. The parents Kali Aus and IR 87707–446-B-B-B showed better performance in term of grain yield and number of nodal roots in both wet and dry seasons, whereas NSICRc 222 and Vandana showed better grain yield performance under dry season (Table 3). Similarly, NSICRc 222 and NSICRc 192 were having higher root hair length and density and flag leaf area compared to other parents. In term of SPAD and LCC, IR 87707-446-B-B-B and Vandana showed better performance. The trend of change of LCC and SPAD value across the progenies was almost similar indicating LCC (which is cheap to buy for farmers) is a good alternative to SPAD (which is expensive for farmers to buy) to decide on the time and dose of fertilizer application under DDSR. The greater radiations during grain filling stage in dry season contributing to the higher grain yield of dry season experiment compared to wet season experiment. The 1000 grain weight was found to be higher for IR 87707-446-B-B-B and NSICRc 192 across seasons. Transgressive segregants were observed for different seedling establishment, root morphological, plant morphological, nutrient uptake, grain yield and yield contributing traits. The traits such as SY and GY (except 2016DS) showed normal distribution. Positive skewness was observed for DTFirst emergence, DTFull emergence, NR, RHL, RHD, BS, FLAngle, SPAD, PHT, DTF and N, P, Fe, Zn uptake. The traits FLA, CD and SD exhibited negative skewness.

**Table 3:** Mean of the MAGIC population and five parents for different traits in 2015WS, 2016DS and combined seasons under the dry direct seeded cultivation conditions

Traits		NR1	NR2	NR3	RHL	RHD	LCC	SPAD	FLL	FLW	FLA	FLAngle	DTFirst	DTFull	SD	CD	BS	BM	PHT	DTF	BMF	VVG	NPT	PL	FG/P	1000GW	SY	GY
Population mean	2015WS	10	13	24	2	2	2.8	37.5	26.9	1.4	28.2	38.3	5	7	5.0	4.8	0.3	1.7	112	88	8245	3	10	25.4	107	25.6	5541	3769
	2016DS	8	22	30	2	2	3.0	37.9	26.6	1.2	24.1	44.2	6	9	6.1	4.5	0.7	3.6	99	77	6462	4	9	23.0	116	27.1	4668	4472
	Combined	9	18	27	1	1	2.9	37.9	26.2	1.1	26.2	41.8	5	8	5.6	4.6	0.5	2.6	106	82	7344	4	10	24.2	112	26.4	5086	4059
NSICRc 222	2015WS	10	12	30	2	2	2.8	36.4	29.5	1.3	28.0	38.3	4	6	4.7	4.6	0.4	2.2	98	97	8209	3	10	25.2	121	24.6	4210	3382
	2016DS	11	21	22	2	2	3.2	38.8	27.6	1.1	22.9	43.5	5	10	5.0	4.1	0.8	4.0	93	81	6583	4	11	23.6	152	24.5	3866	4952
	Combined	10	16	26	2	2	3.0	37.6	28.8	1.2	25.6	40.9	5	8	4.8	4.3	0.6	3.1	96	89	7335	4	10	24.4	137	24.5	4038	4199
IR 87707-446-B-B-B	2015WS	11	16	28	1	1	3.0	38.4	24.8	1.3	24.1	40.0	5	6	4.9	4.6	0.3	1.7	113	85	8833	3	10	26.3	100	28.0	6319	3954
	2016DS	8	26	27	2	1	2.9	39.1	25.5	1.1	21.7	33.5	5	7	6.3	3.9	0.8	3.9	98	73	5778	4	12	25.5	100	30.2	6346	4474
	Combined	10	21	27	1	1	3.0	38.6	24.8	1.1	22.9	36.7	5	7	5.6	4.2	0.6	2.8	105	79	7750	3	11	25.9	100	29.1	6333	4157
NSICRc 192	2015WS	10	12	21	2	2	2.6	35.5	25.7	1.4	27.0	31.7	5	7	4.5	4.7	0.3	1.4	110	86	6389	4	9	25.3	103	24.3	3709	2352
	2016DS	7	19	24	2	2	2.9	36.9	32.7	1.3	30.8	45.0	6	10	6.0	4.6	0.3	1.5	102	71	7715	5	6	24.4	126	28.2	3468	3944
	Combined	9	15	22	2	2	2.8	36.2	29.4	1.3	29.1	38.2	5	8	5.3	4.6	0.3	1.4	106	79	7478	5	8	24.8	114	26.2	3589	3091
Vandana	2015WS	11	11	23	1	1	2.6	37.5	26.9	1.4	27.7	28.0	5	7	4.0	5.1	0.3	1.7	107	85	8306	5	9	25.8	124	22.8	3868	2950
	2016DS	11	21	21	1	1	3.0	38.0	23.7	1.1	19.2	36.5	5	8	6.0	4.1	0.1	0.7	96	71	4346	4	9	23.4	125	24.6	4248	4329
	Combined	11	16	22	1	1	2.8	37.7	25.6	1.2	23.5	32.1	5	7	5.0	4.6	0.2	1.2	101	78	6439	4	9	24.6	124	23.7	4058	3575
Kali Aus	2015WS	11	14	29	1	1	2.2	33.2	24.8	1.3	23.6	42.9	4	6	3.8	4.0	0.1	0.5	111	76	10530	1	11	22.0	82	23.9	5010	4532
	2016DS	10	24	26	1	1	2.5	34.3	21.4	1.0	16.8	23.5	5	7	4.0	3.1	0.1	0.5	85	67	4398	3	12	18.1	64	26.8	4618	4160
	Combined	10	19	27	1	1	2.3	33.7	22.8	1.2	19.9	33.2	4	7	3.9	3.5	0.1	0.5	98	71	7184	2	11	20.0	73	25.3	4814	4301
CV	Combined	16.2	16.7	17.5	31.8	31.5	9.0	9.1	12.8	15.5	13.1	22.4	8.5	10.8	11	8.7	35.0	32.0	8.3	5.4	19.4	24	15.8	5.6	16.2	8.0	18	18.7
P (F-test)	2015WS	**	**	*	*	**	**	*	NS	*	*	*	*	*	*	*	*	*	***	***	NS	*	NS	NS	NS	***	*	*
	2016DS	**	NS	*	*	*	***	*	**	**	***	**	*	***	***	***	***	***	***	***	**	**	***	***	***	***	***	***
Heritability	2015WS	0.63	0.58	0.43	0.19	0.25	0.59	0.45	0.21	0.22	0.20	0.25	0.10	0.12	0.20	0.30	0.20	0.20	0.58	0.80	0.18	0.37	0.01	0.30	0.00	0.79	0.55	0.32
	2016DS	0.12	0.17	0.20	0.20	0.12	0.37	0.19	0.54	0.52	0.44	0.47	0.14	0.31	0.40	0.60	0.50	0.50	0.82	0.91	0.39	0.29	0.44	0.43	0.31	0.40	0.49	0.58

*NR1* number of nodal roots at 15 DAS, *NR2* number of nodal roots at 22 DAS, *NR3* number of nodal roots at 29 DAS, *RHL* root hair length, *RHD* root hair density, *LCC* leaf color chart, *SPAD* cholorophyll content, *FLL* flag leaf length (cm), *FLW* flag leaf width (cm), *FLA* flag leaf area (cm^2^), *FLAngle* flag leaf angle, *DTFirst* days to first emergence (days), *DTFull* days to full emergence (days), *SD* stem diameter (mm), *CD* culm diameter (mm), *BS* bending strength (kg cm), *BM* bending moment (kg cm^2^), *PHT* plant height (cm), *DTF* days to 50% flowering (days), *BMF* biomass at 50% flowering (g), *VVG* vegetative vigor score, *NPT* number of productive tillers per plant, *PL* panicle length (cm), *NFG/P* number of filled grains/panicle, *1000GW* 1000 grain weight (g), *SY* straw yield (kg ha^−1^), *GY* grain yield (kg ha^−1^)

NS non-significant, *significant at < 0.05 level, **significant at < 0.01 level, *significant at < 0.001 level

**Fig. 2:**
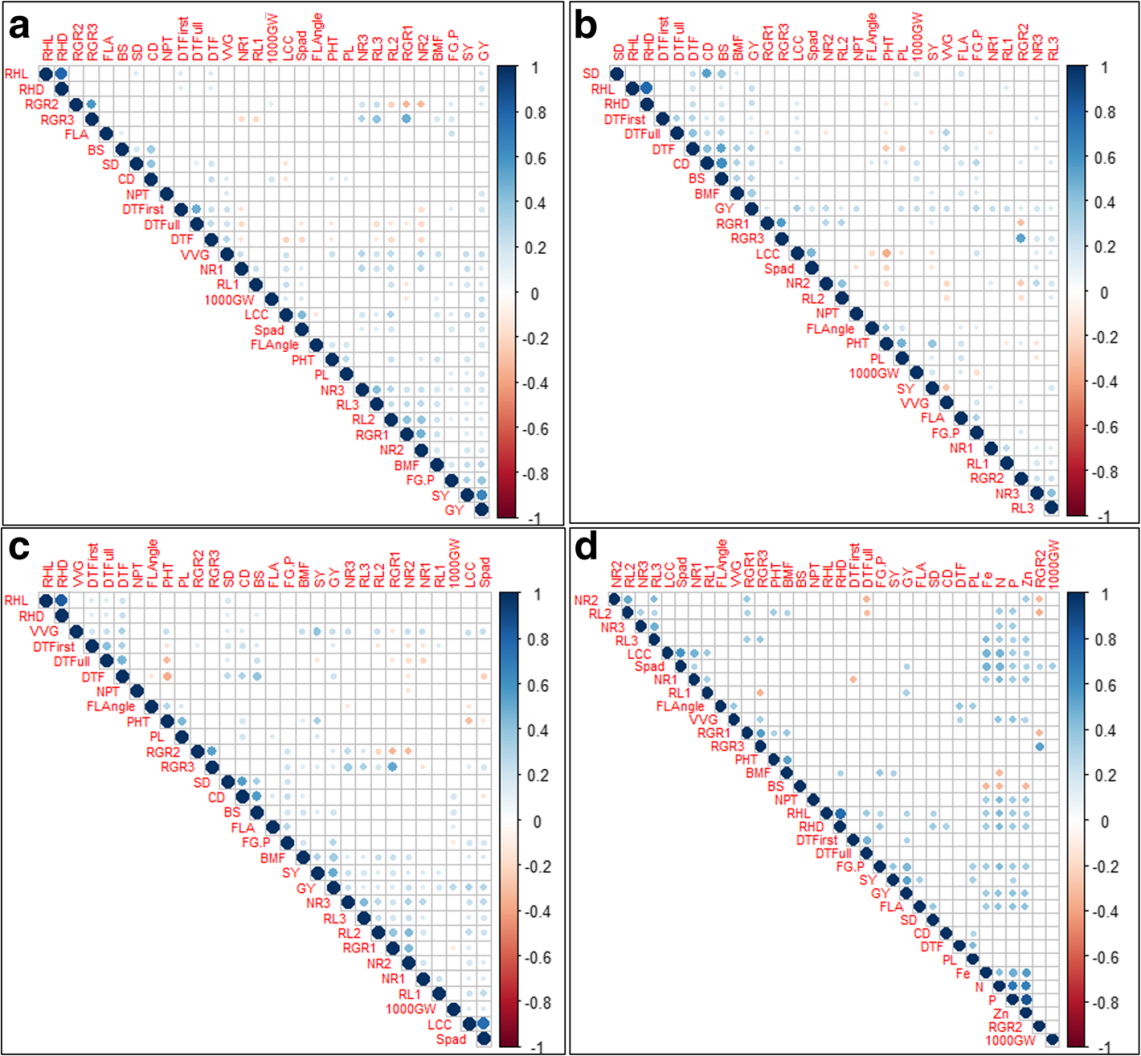
Phenotype-Phenotype correlation plot of different seedling establishment, root, grain and grain yield contributing traits considering whole population; **a** in 2015WS. **b** in 2016DS. **c** in combined seasons. **d** different seedling establishment, root, nutrient uptake, grain and grain yield contributing traits considering 60 progenies (30 high yielding and 30 low grain yielding used for nutrient uptake analysis). The blue color indicates the significant positive correlation and red color indicates the significant negative correlation among different traits. *RGR1: relative growth rate from 22 to 15 DAS, RGR2: relative growth rate from 29 to 22 DAS, RGR3: relative growth rate from 29 to 22 DAS, NR1: number of nodal roots at 15 DAS, NR2: number of nodal roots at 22 DAS, NR3: number of nodal roots at 29 DAS, RL1: maximum root length (cm) at 15 DAS, RL2: maximum root length (cm) at 22 DAS, RL3: maximum root length (cm) at 29 DAS, RHL: root hair length, RHD: root hair density, LCC: leaf color chart, SPAD: cholorophyll content, FLL: flag leaf length, FLW: flag leaf width, FLA: flag leaf area, FLAngle: Flag leaf Angle, DTFirst: days to first emergence, DTFull: days to full emergence, SD: stem diameter, CD: culm diameter, BS: bending strength (kg cm), BM: bending moment (kg cm*^*2*^*), PHT: plant height (cm), DTF: days to 50% flowering (days), BMF: biomass at 50% flowering (g), VVG: vegetative vigor score, NPT: number of productive tillers per plant, PL: panicle length (cm), NFG/P: number of filled grains/panicle, 1000GW: 1000 grain weight (g), SY: straw yield (kg ha*^*− 1*^*), GY: grain yield (kg ha*^*− 1*^*), N: nitrogen, P: phosphorus, Fe: iron, Zn: zinc*

The grain yield was significantly and positively correlated with seedling establishment traits (vegetative vigor, days to first and full emergence), root morphological and nutrient uptake related traits (nodal root, root hair length, density, LCC, SPAD) and grain yield attributing traits (number of productive tillers per plant, number of filled grains/panicle, 1000 grain weight) in 2015WS (Fig. 2a), 2016DS (Fig. 2b) and combined seasons (Fig. 2c) analysis.

Strong seedling vigor and early and uniform emergence are desirable traits for enhancing crop establishment and weed competitiveness under DDSR. Early vigor and uniform emergence are complex traits which are influenced by different environmental and soil factors. Across the seasons, significant variability was observed among the progenies for DTFirst and DTFull emergence. The existing variations could be exploited in identifying the favourable alleles to be further used in marker-assisted introgression program. In the present study, direct significant positive correlation of grain yield with seedling establishment traits and grain yield attributing traits indicated the complexity of the relationship of these traits from seedling to reproductive stage in improving grain yield under DDSR. Investigating the genetic basis and relatedness of these traits and using them in marker-assisted breeding program is the urgent need of DDSR varietal development program.

The nutrient uptake (N, P, K and Zn) was significantly and positively correlated with nodal root, root length, root hair density, LCC, SPAD, number of productive tillers per plant, number of filled grains/panicle and grain yield (Fig. 2d). The significant positive correlation of nutrient uptake with grain yield and root morphological traits indicated the role of root traits in water-nutrient uptake and their effect in improving grain yield under DDSR. The significant positive correlation of a number of nodal roots at 15 DAS (seedling stage) with nitrogen, phosphorus, iron and zinc and at later stage 22 DAS with zinc uptake and at 29 DAS with nitrogen and phosphorus uptake (Fig. 2) indicated the importance of nodal root at each growth stage. Across the seasons, although the direction of correlation (whether positive or negative) for the seedling establishment, root and grain yield attributing traits such as DTFull emergence, nodal root, maximum root length, LCC, SPAD, FLAngle, CD, DTF, NPT and SY with GY was same but the significance level was different. To investigate the underlying relationship among the proxy traits for high yield and better water-nutrient uptake under DDSR, the traits data were also analyzed with PCA. This partitioned the total variations into correlated (RGR, NR, RHD, LCC, BS, SY) and inversely correlated traits (DTFirst, DTFull and DTF) with GY (Additional file 2: Figure S4). Some traits such as RHD, RHL, LCC, SD, CD, BS, PHT and FLAngle showed the effect of seasonal variability. This highlighted the role and phenotypic plasticity behaviour of different traits in increasing yield across seasons. Therefore, a better understanding of the relationship between root morphological traits at different time points, across different seasons and at different developmental stages with water-nutrient uptake is likely to provide a novel dimension in selecting traits and high-yielding varieties needed for DDSR. The correlation among the traits across seasons is shown in Additional file 1: Table S1.

### Population structure and linkage disequilibrium (LD)

**Fig. 3:**
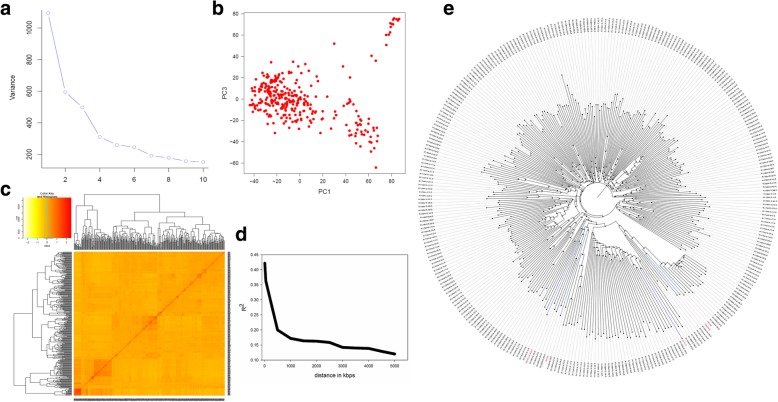
Genetic relatedness and population structure of MAGIC population association panel. **a** Scree plot showing most of the variability explained by first two PCs for association study. **b** Variation of first two principal components. **c** Kinshi*p* values. **d** Average LD as a function of inter-SNP markers distance estimated in the MAGIC population association panel. **e** Phylogenetic neighbor-joining tree of MAGIC population used for phenotyping under dry direct seeded cultivation conditions

Population structure and LD decay were estimated using the genetic structure of the 300 progenies from MAGIC population and 5 parents using 24,306 SNPs distributed across 12 rice chromosomes. The scree plot produced from GAPIT showed that first four principal components were informative, and then there was a gradual decrease (Fig. 3a) until the tenth PC component. The PC1 explained 32.7%, PC2 explained 15.7%, PC3 explained 13.2% and PC4 explained 8.5% of total variance (Fig. 3b). All the progenies were divided into two distinct major groups and further subdivided into subgroups (Fig. 3c). The divergent *aus* and *indica* subpopulations of rice are adapted to different ecologies and geographies, and also harbor different traits of interest and alleles. The *aus* subpopulation is of interest as it has important source of alleles for drought tolerance and root traits while *indica* subpopulation harbors huge genetic variations. The kinship heatmap showed that most of the kinship value concentrated at *0.0 level indicating a very weak relatedness in the MAGIC population derived GWAS panel (Fig. 3c). Pairwise linkage disequilibrium (r^2^) was calculated between all 24,306 markers. The average value of r^2^ as a function of the inter-marker distance was used for LD decay calculation. The average r^2^ for close markers of 5 kbs starts at 0.4, and the LD (r^2^) dropped to half of its maximum value (0.2) at around 500 kb (Fig. 3d). The phylogenetic structure of the MAGIC population panel was illustrated using unweighted NJ tree (Fig. 3e).

### Genome wide association study

**Fig. 4:**
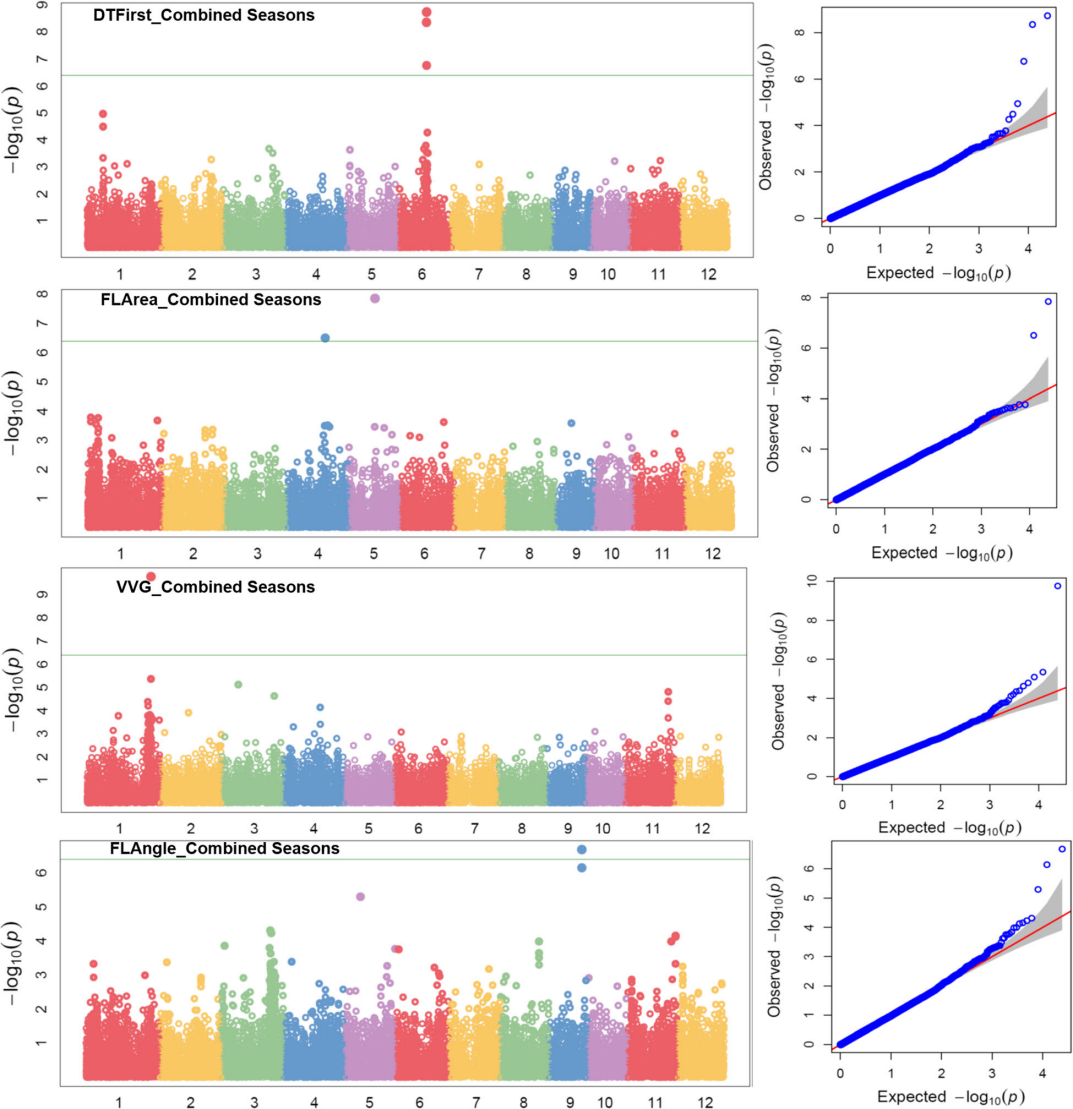
Manhattan and Q-Q plots of genome wide association mapping of seedling establishment traits (DTFirst: days to first emergence, VVG: vegetative vigor), plant morphological traits (FLA: flag leaf area, FLAngle: flag leaf angle) across seasons under dry direct seeded cultivation conditions. The y axis in each graph represent −log_10_P for the *P* value of the MTAs, while linkage groups are indicated on the x axis

**Fig. 5:**
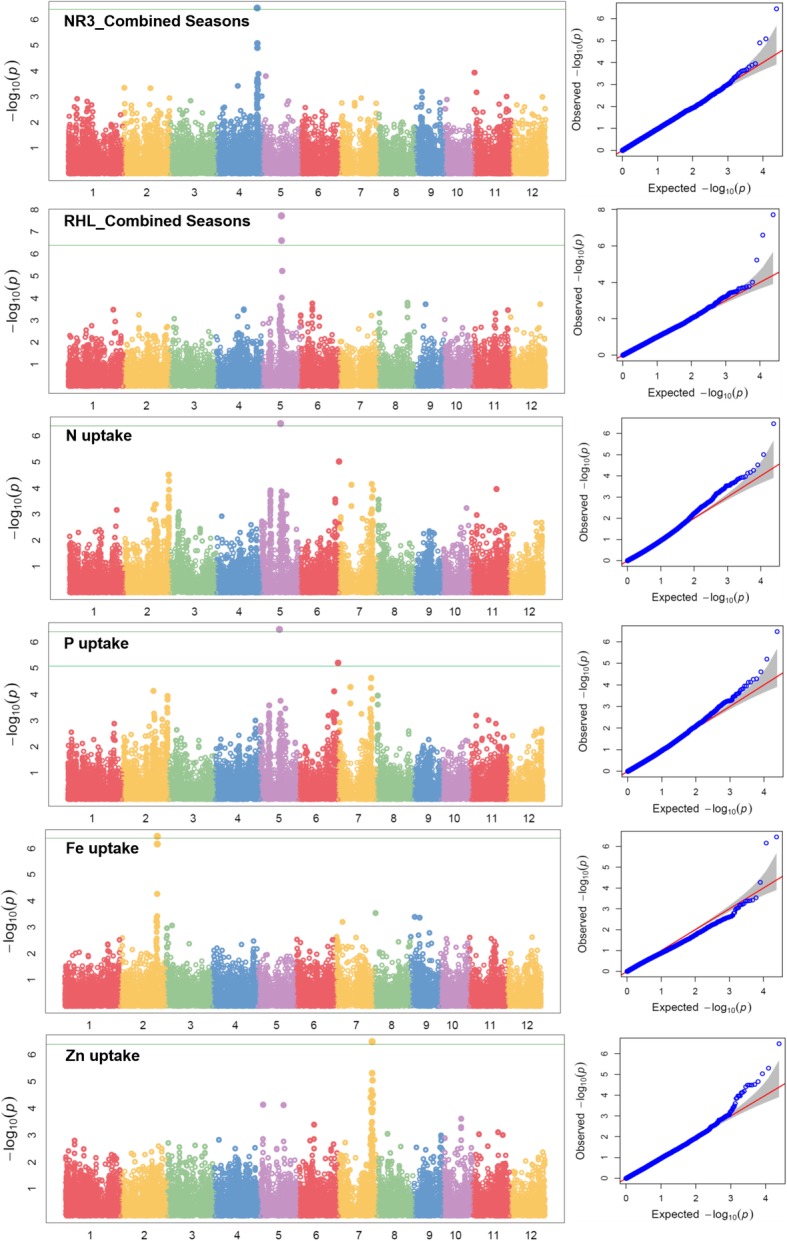
Manhattan and Q-Q plots of genome wide association mapping of root traits (NR3: number of nodal root at 29 days after seeding, RHL: root hair length) across seasons and nutrient uptake (N: nitrogen, P: phosphorus, Fe: iron, Zn: zinc) under direct seeded cultivation conditions. The y axis in each graph represent −log_10_P for the P value of the MTAs, while linkage groups are indicated on the x axis. Note: Similar SNPs were detected in 2015WS and 2016DS

**Fig. 6:**
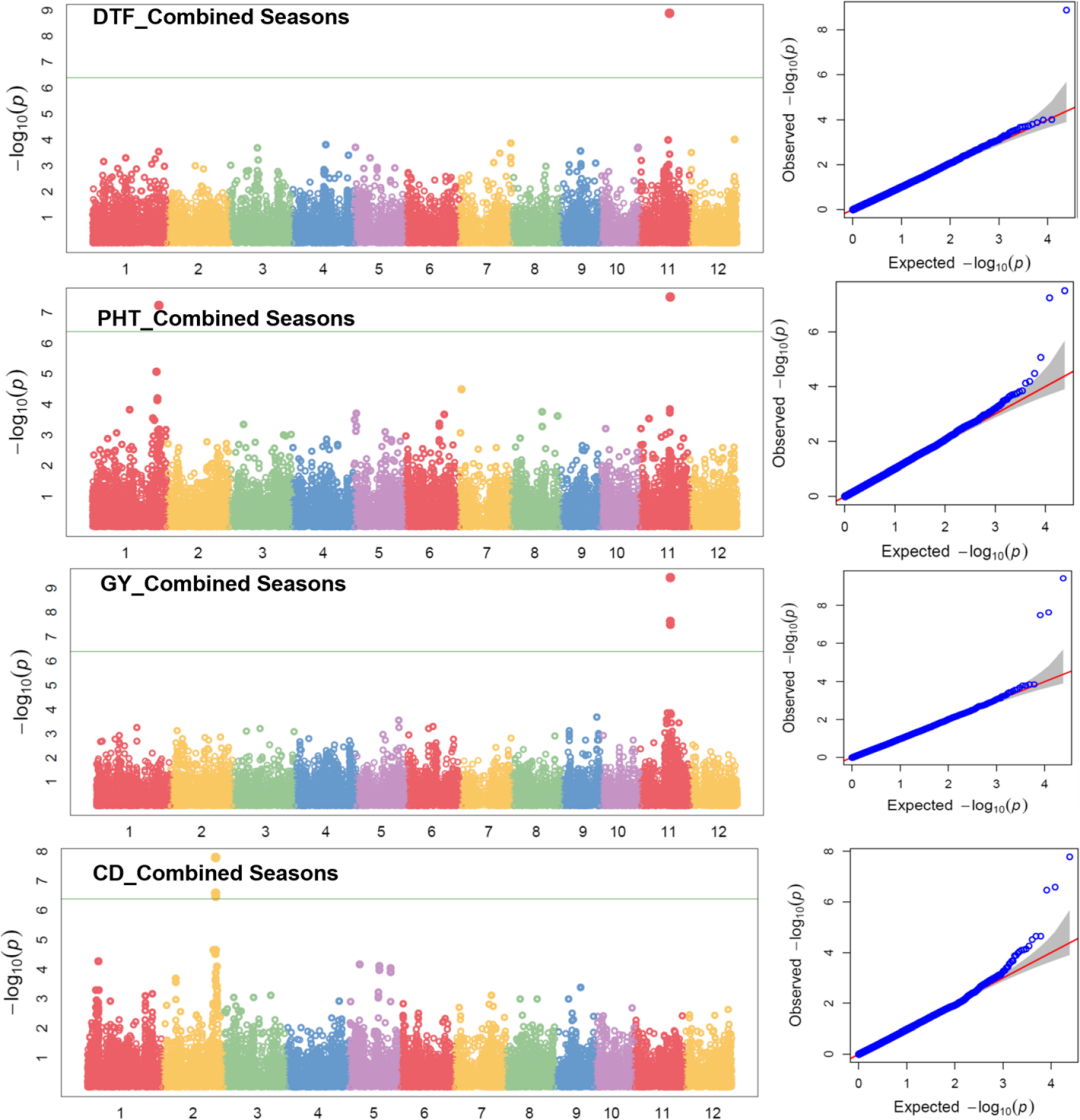
Manhattan and Q-Q plots of genome wide association mapping of agronomic traits (DTF: days to 50% flowering, PHT: plant height, GY: grain yield) and lodging resistance traits (CD: culm diameter) across seasons under direct seeded cultivation conditions. The y axis in each graph represent −log_10_P for the P value of the MTAs, while linkage groups are indicated on the x axis

To take advantage of identified marker-trait associations directly in MAS (marker-assisted selection), GWAS was performed directly on the MAGIC population. The high phenotypic variability existing in the MAGIC population coupled with high marker density across all chromosomes provided a strong base to the whole genome association mapping. About 25 and 12.5% of the genotypes in F_3_ and F_4_ populations were heterozygous. A total of 2,75,586 SNPs were called from the GBS data. After filtering SNP with call rate 80% and MAF (minor allele frequency) of > 5%, a total of 24,306 polymorphic SNPs were retained for further GWAS. The information’s on *p*-values, FDR, SNP, SNP position and major and minor alleles are shown in Table 4. The Manhattan plots depicting -log (p-values) and Q-Q plots (quantile-quantile) showing the expected vs. observed p-values for the SNP-based progeny-trait of interest associations are reported in Fig. 4 (seedling establishment traits and plant morphological traits), Fig. 5 (root morphological traits and nutrient uptake) and Fig. 6 (agronomic traits, grain yield, and lodging resistance traits).

**Table 4:** Identified significant SNPs-traits associations in genome wide study on the MAGIC population

Traits	SNP	Chr	Position	QTL span (kb)	2015WS			2016DS			Source: http://qtaro.abr.affrc.go.jp	
					*p*-value	R^2^ (%)	FDR	*p*-value	R^2^ (%)	FDR	Gene	QTLs
NR3	S4_31728342	4	31728342		2.06 × 10^− 7^	9	0.0050	2.28 × 10^− 7^	16	0.00552	*nal1*(*nal5*) [48]	*rfw4a* [49], Root thickness [50, 51], Rooting depth [50]
RHL	S5_15470847	5	15470847	0.033^a^	1.99 × 10^− 7^	11	0.0048	5.20 × 10^−8^	12	0.00126	*OsIPT3* [52]; *OsEXPA3* [53]	
	S5_15470880	5	15470880		4.31 × 10^−6^	9	0.0523	6.79 × 10^− 7^	10	0.00823		
RHD	S5_15470880	5	15470880		4.34 × 10^−6^	12	0.0603	2.04 × 10^− 7^	11	0.00495	*OsIPT3* [52]; *OsEXPA3* [531]	
FLA	S5_15133031	5	15133031		2.88 × 10^− 8^	19	0.0007	1.50 × 10^− 7^	18	0.00357		
	S4_21832119	4	21832119		4.75 × 10^− 6^	16	0.0575	2.95 × 10^− 7^	18	0.00357	*tdd1* [54]	
FLAngle	S9_19178976	9	19178976		1.82 × 10^− 7^	12	0.0044	2.99 × 10^− 7^	13	0.00725	*TAC1* [55]	*Ta1* [56]*, Ta* [57]
DTFirst	S6_16444037	6	16444037	0.045^a^	2.04 × 10^− 7^	18	0.0032	3.40 × 10^− 7^	19	0.00824		*qGP-6* [58]
	S6_16444038	6	16444038		2.62 × 10^− 7^	18	0.0032	1.00 × 10^− 6^	19	0.01212		
	S6_16444082	6	16444082									
DTFull	S11_24806601	11	24806601	0.036^a^	1.67 × 10^− 7^	15	0.0040					*qGP-11, qGI-11* [58], *yld11.1, gpl11.1, gw11.1* [59]
	S11_24806565	11	24806565					2.47 × 10^−7^	20	0.00600		
	S11_24806596	11	24806596		3.41 × 10^−7^	15	0.0041	2.84 × 10^−6^	18	0.03302		
Spad	S2_16191048	2	16191048	177	2.43 × 10^−7^	14	0.0059	5.18 × 10^−9^	18	0.00013	*OsWRKY42* [60]	*Ghd7* [61]
	S2_16013833	2	16013833					1.26 × 10^− 6^	14	0.01523		
BS	S3_21737519	3	21737519		9.39 × 10^−8^	12	0.0023	2.76 × 10^−7^	19	0.00669		
SD	S2_31078747	2	31078747		3.69 × 10^−7^	12	0.0089	2.47 × 10^− 7^	17	0.00599	*bc3* [62]	
CD	S2_30984762	2	30984762	93.99	9.97 × 10^− 7^	13	0.0024	2.83 × 10^− 7^	27	0.00686	*bc3* [62]	
	S2_30987475	2	30987475		1.03 × 10^−6^	12	0.0125					
	S2_31078747	2	31078747		1.26 × 10^−7^	10	0.1020					
PHT	S11_17805610	11	17805610		1.88 × 10^−7^	26	0.0035	1.77 × 10^− 7^	29	0.00260		
	S1_38481437	1	38481437		2.87 × 10^−7^	26	0.0035	2.15 × 10^− 7^	29	0.00260	*Sd1* [63], *qDTY*_*1.1*_ [64]	
GY	S11_17412133	11	17412133	0.005^a^	2.12 × 10^− 7^	26	0.0051	3.02 × 10^− 7^	26	0.00073		*yld11.1*, *gpl11.1*, *gw11.1* [59]
	S11_17412134	11	17412134		4.11 × 10^−6^	24	0.0498	1.08 × 10^−6^	24	0.00869		
	S11_17412139	11	17412139		6.80 × 10^−6^	24	0.0549	2.91 × 10^−7^	24	0.00352		
SY	S11_15708717	11	15708717		3.15 × 10^−7^	17	0.0076	2.20 × 10^−7^	15	0.00534		
DTF	S11_17316419	11	17316419		7.71 × 10^−8^	24	0.0019	1.87 × 10^−8^	30	0.00045		
VVG	S1_37653811	1	37653811	14^a^	1.52 × 10^− 8^	17	0.0004	2.23 × 10^− 8^	15	0.00054	*Lax* [65], *moc* [66]	
	S1_37639734	1	37639734		1.70 × 10^−7^	15	0.0021					
	S11_25462225	11	25462225					2.68 × 10^−7^	13	0.00325		
Fe uptake	S2_29221003	2	29221003	0.012 ^a^	3.49 × 10^−7^	86	0.0083				*OsYSL15* [67], *OsCKI1* [68], *OsGPX3* [69], *MAIF1* [70]	
	S2_29221015	2	29221015		6.84 × 10^− 7^	81	0.0083					
N uptake	S5_14987295	5	14987295		3.43 × 10^−7^	90	0.0083				*OsIPT3* [52]	
	S6_30887442	6	30887442		9.82 × 10^−6^	72	0.1190				*OsPTR9* [71]	
P uptake	S5_14987295	5	14987295		3.36 × 10^−7^	92	0.0081				*OsIPT3* [52]	
	S6_27868961	6	27868961	3018	7.64 × 10^−5^	71	0.2644				*OsPTR9* [71]	
	S6_30887442	6	30887442		6.45 × 10^−6^	80	0.0781					
Zn uptake	S7_26042045	7	26042045		3.29 × 10^−7^	90	0.0080				*OsNAS3* [72], *aldolase* [73]	*7–3* [74]

*NR3* number of nodal roots at 29 DAS, *RHL* root hair length, *RHD* root hair density, *FLA* flag leaf area, *FLAngle* Flag leaf angle, *DTFirst* days to first emergence, *DTFull* days to full emergence, *SPAD* cholorophyll content, *BS* bending strength, *SD* stem diameter, *CD* culm diameter, *PHT* plant height, *GY* grain yield, *SY* straw yield, *DTF* days to 50% flowering, *VVG* vegetative vigor, *Fe uptake* iron uptake, *N uptake* nitrogen uptake, *P uptake* phosphorus uptake, *Zn uptake* zinc uptake, *DS* dry season, *WS* wet season, *R*^*2*^ percent phenotypic variance (PV) explained by SNPs, *FDR* false discovery rate

^a^The associated SNPs with very small interval (≤14 kb) can be considered as the same locus

### Significant MTAs for molecular breeding for DDSR

The main objective of the present study was to identify the significant MTAs for the traits related to improving grain yield and adaptability of progenies under DDSR. A total of 37 significant trait-SNP associations were identified for 20 traits. The percent phenotypic variance (PV) explained by the SNP ranged from 9 to 92% for all the traits measured in the present study. The FDR ranged from 0.264 to 3.69 × 10^− 4^, 0.0330 to 1.25 × 10^− 4^, 0.0534 to 4.60 × 10^− 6^ was observed in 2015WS, 2016DS and combined seasons analysis respectively (Table 4). Among the 37-significant marker-trait associations, 15 were located in the proximity of earlier identified candidate genes (http://qtaro.abr.affrc.go.jp) (Additional file 1: Table S2). The SNP-trait association with lowest p-values were reported for S2_16191048 on chromosome 2 for SPAD (p-value = 5.18 × 10^− 9^ in 2016DS and 1.90 × 10^− 10^ in combined seasons). A total of 12 SNPs reported being associated with root morphological and nutrient uptake traits whereas 5 SNPs reported to be associated with grain yield and yield contributing traits. Five SNPs observed to be associated with morphological traits (FLA, FLAngle, PHT). The plant height related SNP S1_38481437 on chromosome 1 colocalized with rice semi-dwarfing gene (*Sd1*; [63, 64] which encodes a mutant enzyme Gibberellin 20-Oxidase, involved in the synthesis of gibberellin and QTL *qPHT*_*1.1*_ [19]. Similarly, five SNPs reported to be significantly associated with lodging resistance traits (BS, SD, CD) and nine SNPs with seedling establishment traits (DTFirst, DTFull, VVG). S2_31078747 and S2_30984762 on chromosome 2 significantly associated with stem and culm diameter respectively, found to be colocated with Rice BRITTLE CULM 3 (*BC3*) gene which is known to be involved in secondary cell wall synthesis [62]. A significant association signal associated with vegetative vigor was detected for 14 kb region span between SNPs S1_37639734 and S1_37653811. This region was located in proximity to *lax* [65] and *moc2* [66] candidate genes controlling shoot branching and tiller growth respectively.

The SNP S2_31078747 (SD, CD), S5_14987295 (N, P uptake) and S5_15470880 (RHL, RHD) observed to be significantly associated with more than one trait. For SNPs, S5_15470847 and S5_15470880 for root hair length and root hair density the colocalization was found with genes *OsIPT3* [52] and *OsEXPA3* [53] which were reported to betightly linked with the root growth in rice. *OsEXPA3* gene reported to be required for the development of primary root length, lateral root density, root vascular bundle cell length and growth in rice. In addition, these identified SNPs also located in the QTL region associated with root hair density as reported by Sandhu et al. [19]. Three SNPs S11_17412133, S11_17412134 and S11_17412139 on chromosome 11 significantly associated with grain yield located in the previously identified QTLs (*yld11.1*, *gpl11.1*, *gw11.1*, 17246592–23651853 bp) region. This region was reported to play an important role in improving grain yield in an *Oryza sativa* × *Oryza rufipogon* BC_2_F_2_ population under upland conditions [59].

A 0.483 Mb region on chromosome 5 found to be associated with N, P uptake, FLA, RHL and RHD and 0.489 Mb region on chromosome 11 with DTF, PHT, and grain yield. A 0.655 Mb region on chromosome 11 reported being associated with seedling establishment traits (DTFull and VVG). For most of the traits like DTFirst, DTFull, CD, GY and VVG, the identified significant multiple SNPs which were present in the full LD region on the same chromosome were having almost the same level of significance across seasons. Some SNPs associated with more than one trait such as S5_14987295 (N, P uptake), and S5_15470880 (RHL, RHD) were having the same level of significance in 2015WS, 2016DS and combined seasons. The colocation of SNPs related to nutrient uptake and root traits further confirmed by the significant positive correlation of root traits with nutrient uptake. The identification of progenies carrying multiple superior haplotype associated with various colocated traits would significantly aid to the development of rice varieties adapted to DDSR. Introgression of superior haplotypes that are responsible for improving grain yield and water-nutrient uptake under DDSR using haplotype-based breeding may open avenues for designing next generation DDSR adapted rice varieties.

The identified significant marker-trait associations on the same chromosome within the 500 kb region, where the LD decays half of its maximum value were assigned as putative QTLs. The associated SNPs with very small interval (≤14 kb) can be considered as the same locus. The putative QTLs for RHL spanning 0.033 kb region on chromosome 5, for DTFull spanning 0.036 kb and for GY spanning 0.005 kb on chromosome 11, for SPAD spanning 177 kb, and for Fe uptake spanning 0.012 kb on chromosome 2, for VVG spanning 14 kb region on chromosome 1, and for DTFirst spanning 0.045 kb (Table 4).

For the number of nodal roots at 29 DAS on chromosome 4: S4_31728342, ~ 115 kb upstream the positional match was identified as *nal1*(*nal5*) gene, NARROW LEAF1 which regulates leaf and adventitious root development in rice [48]. QTLs for root fresh weight, root thickness [48, 50] in upstream region and for rooting depth [51] in the downstream region has been reported. The *p*-values of significant allelic variation for the number of nodal roots at 29DAS were 5.71 × 10^− 5^, 7.13 × 10^− 6^ and 6.267 × 10^− 6^ in 2015WS, 2016DS and combined season analysis.

The p-values of significant variations for all three significant SNPs associated with grain yield QTL ranged from 3.125 × 10^− 5^ to 8.287 × 10^− 8^ across seasons. The p-values representing allelic variation for nutrient uptake were 0.01184 (for S6_30887442), 0.04321 (for S6_30887442) and 0.02327 (for S5_14987295) for P uptake, 0.02449 (S5_14987295) for N uptake and 0.001039 (S7_26042045) for Zn uptake. Two SNPs S2_29221003 and S2_29221015 on chromosome 2 showed significant associations with iron (Fe) uptake under direct seeded cultivation conditions. Interestingly, the genes *OsYSL15*; a root tissue expressed iron-regulated gene for iron uptake at seedling development stage [67], *OsCKI1*; a rice casein kinase I gene associated with root development) [68], *OsGPX3*; mitochondrial glutathione peroxidase *GPX3* essential in root-shoot development) [69], and *MAIF1*; F-box protein gene promotes root growth in rice [70] reported to be colonized with the iron uptake gene mapped in the present study. In the present study, SNP S5_14987295 on chromosome 5 associated with both nitrogen and phosphorus uptake was identified to be colocated with the gene *OsIPT3* which was involved in cytokinin biosynthesis [52] and QTL *qP*_*5.2*_, *qPU*_*5.2*_ [19]. The zinc uptake linked SNP S7_26042045 on chromosome 7 reported to be located in the QTL region associated with root morphology and distribution in differential water-regime under upland conditions [74]. In addition, this SNP was reported to be colocated with *qZn*_*7.1*_; the QTL detected for grain zinc concentration in a rice MAGIC plus population [75] and with QTL for grain breadth in an *indica* MAGIC population [76]. The colocation of QTLs and positive correlation between grain Zn and grain width [75] indicated the importance of grain dimensional traits during selection process.

**Fig. 7:**
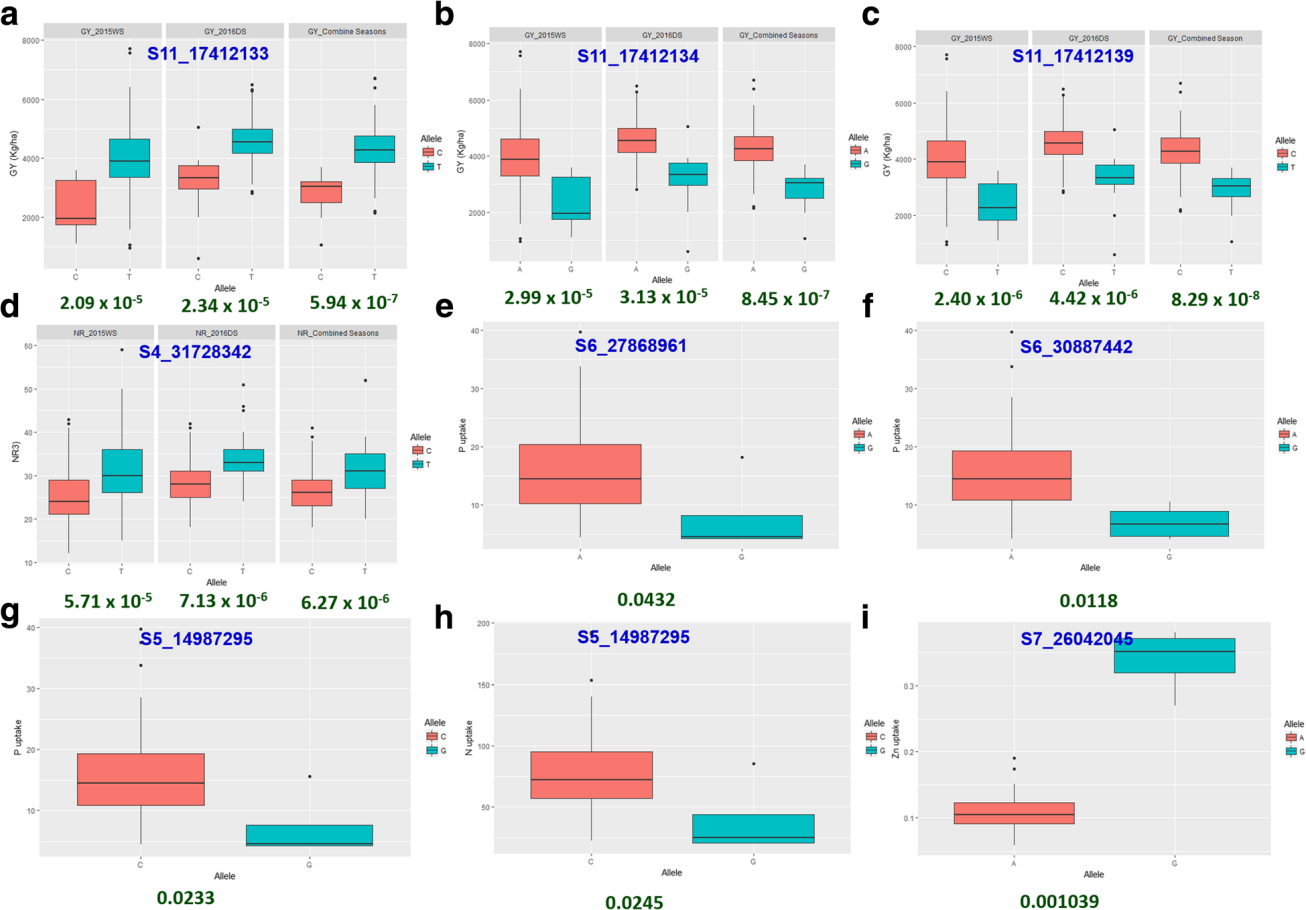
Comparison of allelic effects for the marker-trait associations for; **a, b, c** grain yield. **d** root traits (NR#: number of nodal roots at 29 DAS). **e, f, g** P (phosphorus) uptake. **h** N (nitrogen) uptake. **i** Zn (zinc) uptake. The significant difference (p value in green colour) between the mean values of the allelic class was determined using Kruskal–Wallis test

The allelic effects of the significantly associated markers for root, nutrient uptake and grain yield traits are shown in Fig. 7. The parental allele contributing for the increased grain yield under DDSR were coming from IR 87707–446-B-B-B and Kali Aus parents. The parental allele contributing for increase in nodal roots number and Fe uptake was coming from Kali Aus, for N and P uptake from IR 87707–446-B-B-B and for Zn uptake coming from Vandana.

To identify the candidate genes underlying the significant marker-trait association/putative QTLs MSU v.7 rice genome browser (http://rice.plantbiology.msu.edu/cgi-bin/gbrowse/rice/#search) and the existing literature were searched. The identified candidate genes were in the vicinity of the reported SNPs/putative QTLs in the present study which warrant further investigation and validation. The detailed description of the candidate genes is presented in Additional file 1: Table S2. The LD decays half of its maximum value at 500 kb in the present study and the candidate genes were searched within 120-kb region around, indicating the SNPs have strong LD within the ORF. All these identified significant MTAs and identified the breeding progenies with favourable alleles combinations can be deployed after validation for improving grain yield and adaptability through molecular breeding.

### Performance of selected promising progenies

DDSR technology has huge potential to save water, labor and energy with profitable results along with eco-friendly characteristics avoiding all the penalties entailed with the puddled transplanting system. DDSR offers a choice of rice establishment methods and water requirements for crop establishment and the subsequent crop growth, thus increasing the capacity of poor farmers to cope with the changes induced by the climatic conditions and the reducing soil resources. Farmers can go ahead with direct seeding with minimal soil moisture rather than waiting for sufficient rainfall for transplanting in case of early drought. DDSR also reduces the cost of additional irrigation and the risk of yield loss from the late-season drought. The generations of new DDSR rice varieties would most likely be needed for the sustainable rice production. Seven breeding progenies viz IR 115844-B-32-B, IR 115844-B-349-B, IR 115844-B-326-B, IR 115844-B-366-B, IR 115844-B-316-B, IR 115844-B-69 and IR 115844-B-230 possessing, better root architecture for nutrient uptake and higher yield with combination of favourable allele for the multiple DDSR traits were identified (Table 5).

**Table 5:** Selected promising progenies showing significant variation for nutrient uptake, root traits and grain yield under dry direct seeded cultivation conditions

Designation	N uptake (kg ha^−1^)	P uptake (kg ha^−1^)	Fe uptake (kg ha^−1^)	Zn uptake (kg ha^−1^)	NR at 29 DAS			RHL			RHD			GY (kg ha^−1^)		
	2015WS	2015WS	2015WS	2015WS	2015WS	2016DS	CS	2015WS	2016DS	CS	2015WS	2016DS	CS	2015WS	2016DS	CS
IR 115844-B-32-B	81.71	14.28	3.87	0.151	32	33	33	2	2	2	3	2	2	7708	5713	6710
IR 115844-B-349-B	85.18	23.58	3.06	0.208	33	35	34	2	2	2	2	1	1	7563	5220	6391
IR 115844-B-326-B	46.67	16.18	2.10	0.156	35	37	35	2	3	2	3	2	2	6103	5517	5810
IR 115844-B-366-B	99.95	24.20	2.73	0.190	38	29	33	2	2	2	3	2	2	6405	5054	5729
IR 115844-B-316-B	101.57	17.80	3.70	0.179	31	32	31	1	2	2	2	2	2	5708	5424	5566
IR 115844-B-69	147.09	25.65	3.87	0.227	39	30	35	1	2	2	1	2	2	5495	5401	5401
IR 115844-B-230	113.18	24.95	3.12	0.229	31	28	30	1	2	1	1	1	1	5205	5211	5211
NSICRc 222^†^	65.20	17.50	2.81	0.134	30	22	26	2	2	2	2	2	2	3382	4952	4199
Kali Aus^†^	57.80	12.70	3.57	0.167	29	26	27	1	1	1	1	1	1	4532	4160	4301
NSICRc192^†^	56.40	10.20	1.99	0.127	21	24	22	2	2	2	2	2	2	2352	3944	3091
Vandana^†^	69.70	12.40	3.27	0.184	23	21	22	1	1	1	1	1	1	2950	4329	3575
IR 87707–446-B-B-B^†^	74.10	20.00	2.30	0.139	28	27	27	1	2	1	1	1	1	3954	4474	4157
Mean	83.21	18.29	3.07	0.170	24	30	27	2	2	1	2	2	1	5064	4967	5015
SE mean	8.21	1.55	0.20	0.010	1.61	1.52	1.11	0.14	0.18	0.12	0.23	0.14	0.13	514	169	327
t-test	10.14	11.80	14.98	17.34	15.31	18.57	23.68	10.8	9.33	13.68	8.30	10.42	11.94	9.84	29.38	15.33

^*+*^*Parent, DS* dry season, *WS* wet season, *CS* combined seasons, *NR3* number of nodal roots at 29 DAS, *RHL* root hair length, *RHD* root hair density, *GY* grain yield (kg ha^−1^), *N uptake* nitrogen uptake (kg ha^−1^), *P uptake* phosphorus uptake (kg ha^−1^), *Fe uptake* iron uptake (kg ha^−1^), *Zn uptake* zinc uptake (kg ha^−1^)

The MAGIC population developed in the present study has an advantage over the existing MAGIC population in rice [76] as it has been developed involving upland adapted parents having better root system and improved yield under rainfed environments. The identified promising breeding progenies with favourable alleles in combination for the multiple traits might serve as potential donors for improving grain yield and adaptability under DDSR. The percent improvement in selected promising breeding progenies for nitrogen uptake ranged from 10 to 99% and phosphorus uptake from 18 to 28% over the best performing parent IR 87707-446-B-B-B (Table 5). The percent improvement in the selected progenies for iron uptake ranged from 4 to 8% and for zinc uptake ranged from 3 to 24% over the best performing parents Kali Aus and Vandana, respectively (Table 5). The percent improvement of number of nodal roots at 29 DAS in selected promising progenies varied from 7 to 35%, 8 to 42% and 11 to 30% in 2015WS, 2016DS and combined season, respectively over the best parent Kali Aus across seasons (Table 5). The percent grain yield advantage of selected promising progenies ranged from 15 to 70% in 2015WS, 21 to 37% in 2016DS and 21 to 56% in combined season analysis over the stable yielding parent Kali Aus across seasons (Table 5). The parents NSICRc 192 and NSICRc 222 were superior to other parents for root hair length and root density. Identification of suitable traits and progenies may acclerate the development of DDSR adapted rice varieties.

## Conclusions

A major and successful genome-wide association study was carried out for a set of traits- seedling establishment, root morphological, nutrient uptake, grain yield and yield contributing traits increasing rice yield and adaptation in DDSR. The considerable phenotypic variability observed in the MAGIC population coupled with high marker density across all chromosomes provided a strong case for the success of the whole genome association trait mapping. A total of 37 significant marker-trait associations including nine putative QTLs related to the different DDSR traits were detected. The effectiveness of the identified marker-trait associations/putative QTLs was validated by confirming the involvement of collocated QTLs/candidate genes reported previously. The identified putative QTLs and the promising progenies possessing the identified QTLs may serve as the potential candidate donors to be use in GAB programmes improving grain yield and adaptability of rice under DDSR. Identification of high yielding, resource efficient and direct seeded adapted breeding progenies with favourable alleles for multiple traits may help breeders to recommend for release as varieties to be cultivated by farmers under DDSR.

## Additional files

Additional file 1: **Table S1.** The correlation observed among traits across seasons. **Table S2.** The detailed description on the candidate gene identified near or in the region of significant marker-trait association/putative QTLs identified in the present study using MSU v.7 rice genome browser (http://rice.plantbiology.msu.edu/cgi-bin/gbrowse/rice/#search). (XLSX 73 kb)

Additional file 2: **Figure S1.** Average rainfall data (mm) collected during (a) 2015WS (b) 2016DS. **Figure S2.** The details on the seedling establishment traits, root and nutrient uptake traits, lodging resistance traits, Plant morphological, grain yield and yield attributing traits meausred under the present study. **Figure S3.** Prostrate tester quantifying stem strength at breaking point. **Figure S4.** Principal component analysis plot of phenotypic traits in (a) 2015WS (b) 2016DS and (c) combined season. (DOCX 1984 kb)

## Data Availability

The relevant supplementary data has been provided with the manuscript.
