# Supplementary material for: Genome-wide association study reveals significant genomic regions for improving yield, adaptability of rice under dry direct seeded cultivation condition
Source: BMC Genomics. 2019 Jun 10;20:471. doi: 10.1186/s12864-019-5840-9 (PMC6558851; doi:10.1186/s12864-019-5840-9)
Supplement: Supplementary file 2 — Figure S1. Average rainfall data (mm) collected during (a) 2015WS (b) 2016DS. Figure S2. The details on the seedling establishment traits, root and nutrient uptake traits, lodging resistance traits, Plant morphological, grain yield and yield attributing traits meausred under the present study. Figure S3. Prostrate tester quantifying stem strength at breaking point. Figure S4. Principal component analysis plot of phenotypic traits in (a) 2015WS (b) 2016DS and (c) combined season. (DOCX 1984 kb) [file 12864_2019_5840_MOESM2_ESM.docx]

**Genome-wide association study reveals significant genomic regions for improving yield, adaptability of rice under dry direct seeded cultivation condition**

Sushil Raj Subedi^1,6,7^, Nitika Sandhu^1,8^ , Vikas Kumar Singh^2^, Pallavi Sinha^3^, Santosh Kumar^4^, S P Singh^5^_,_ Surya Kant Ghimire^6^, Madhav Pandey^6^, Ram Baran Yadaw^7^, Rajeev K. Varshney^3^, Arvind Kumar^1*^

1. Rice Breeding Platform, International Rice Research Institute, Metro Manila, Philippines
2. International Rice Research Institute, South Asia Hub, ICRISAT, Patancheru, Hyderabad, India
3. Center of Excellence in Genomics and System Biology, International Crops Research Institute for the Semi-Arid Tropics (ICRISAT), Patancheru, Hyderabad, India
4. ICAR Research Complex for Eastern Region, Patna, Bihar
5. Bihar Agricultural University, Sabour, Bihar
6. Agriculture and Forestry University, **Rampur, Chitwan, Nepal**
7. National Rice Research Program, Hardinath, Nepal
8. Punjab Agricultural University, Ludhiana, India

*Corresponding author: a.kumar@irri.org

*Corresponding author: a.kumar@irri.org

^1^International Rice Research Institute, DAPO Box 7777, Metro Manila, Philippines

**Sushil Raj Subedi** : subedirajsubedi@gmail.com

**Nitika Sandhu** : nitika.sandhu@gmail.com

**Vikas Kumar Singh** : v.k.singh@irri.org

**Pallavi Sinha** : p.sinha@cgiar.org

**Santosh Kumar** : [santosh9239@gmail.com](mailto:santosh9239@gmail.com)

**SP Singh** : [sps2007bau2011@gmail.com](mailto:sps2007bau2011@gmail.com)

**Surya Kant Ghimire** : suryaghimire2003@yahoo.com

**Madav Pandey :** mpandey@afu.edu.np

**Ram Baran Yadaw**  : rbaran_9@yahoo.com

**Rajeev K. Varshney** : [r.k.varshney@cgiar.org](mailto:r.k.varshney@cgiar.org)

**Arvind Kumar** : a.kumar@irri.org

**Corresponding Author*

**Arvind Kumar**

Outcome Theme Leader- Resilient Rice

Plant Breeder, Rice Breeding Platform

International Rice Research Institute, DAPO Box 7777

Metro Manila, Philippines

**Average temperature: 28.3°C**

**Average humidity: 86%**

**Average solar radiation: 14.8 MJ m^-2^**

**Average vapour pressure: 3.1 kPa**

**(b)**

**(a)**

**Average temperature: 32.6°C**

**Average humidity: 83.5%**

**Average solar radiation: 16.2 MJ m^-2^**

**Average vapour pressure: 2.9 kPa**

**Figure S1.** Average rainfall data (mm), temperature, humidity, pressure and air density data collected during (a) 2015WS (b) 2016DS


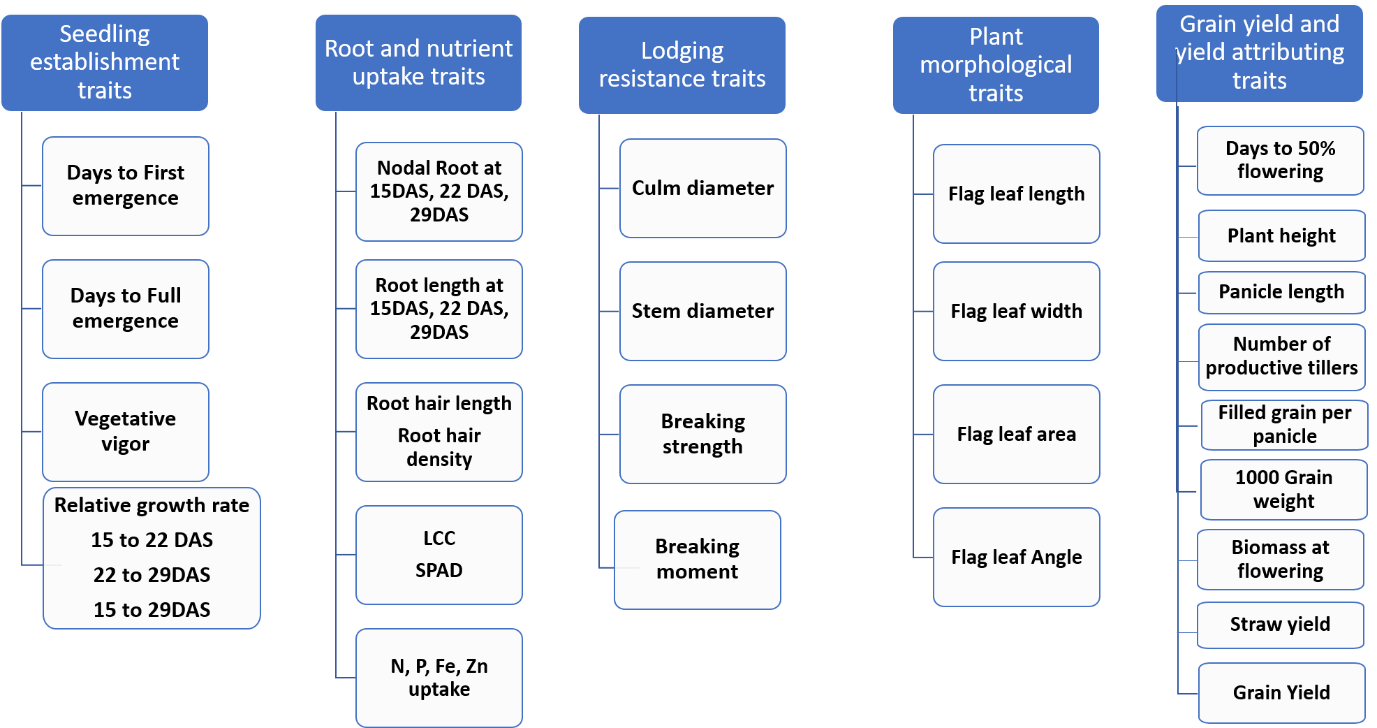


**Figure S2.** The details on the seedling establishment traits, root and nutrient uptake traits, lodging resistance traits, plant morphological, grain yield and yield attributing traits meausred under the present study


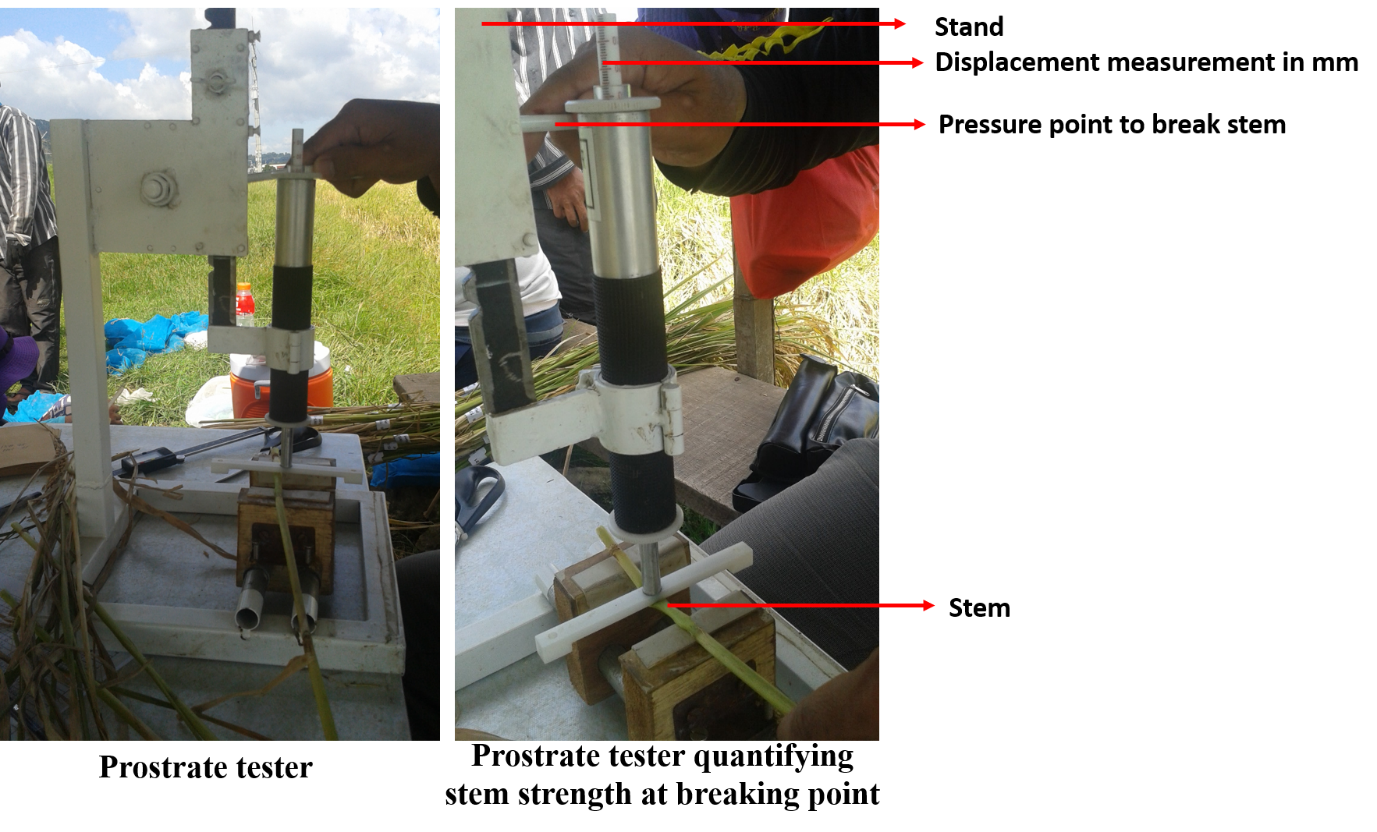


**Figure S3.** Prostrate tester quantifying stem strength at breaking point

**
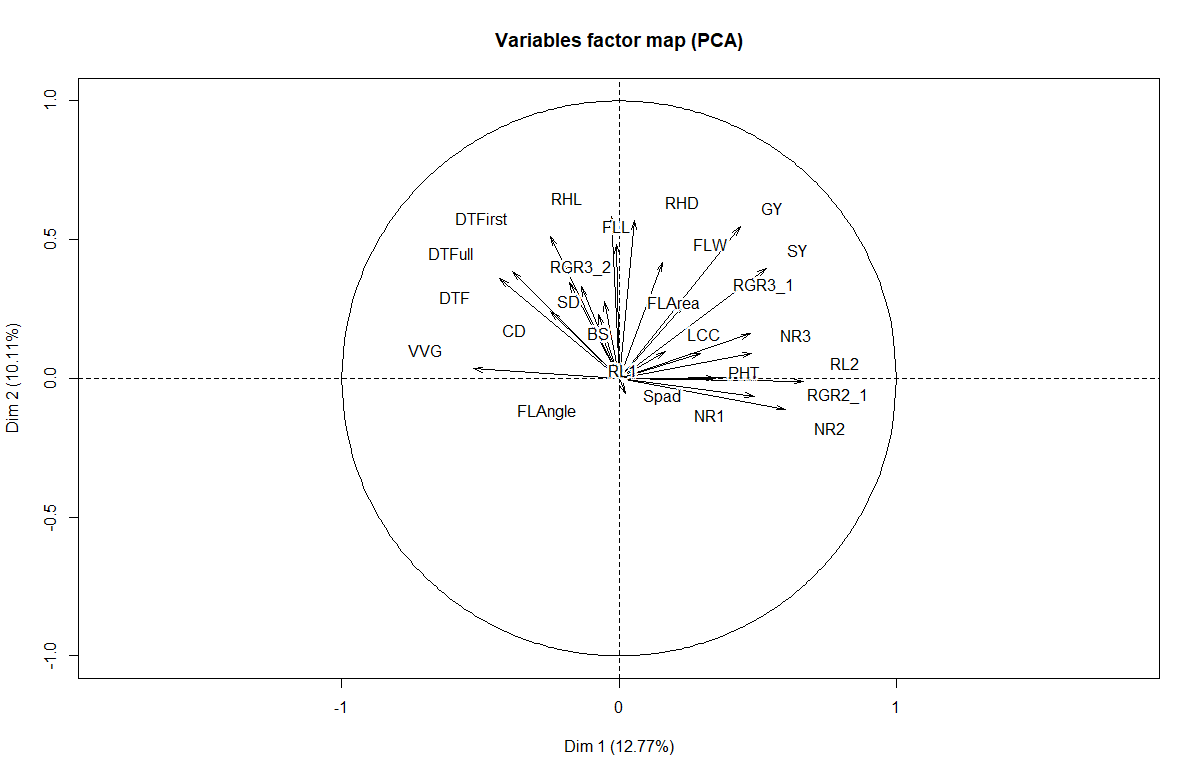
**

**(b)**

**(a)**

**
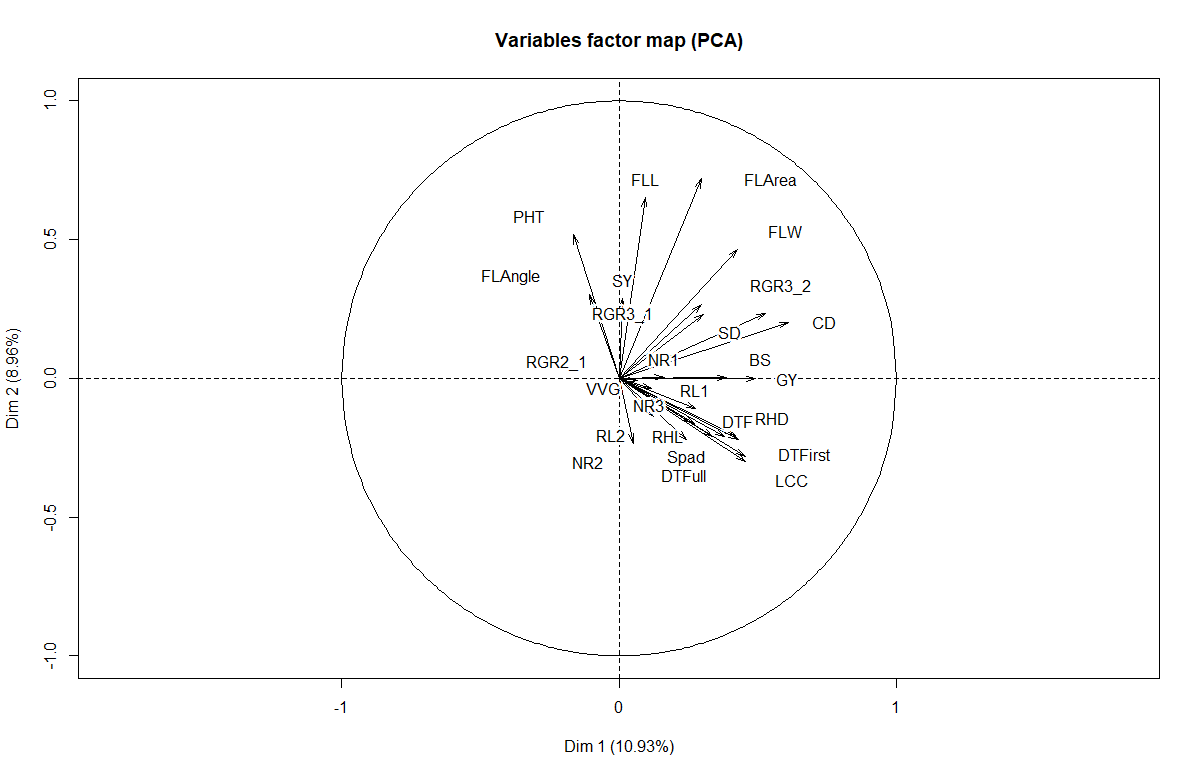
**

**
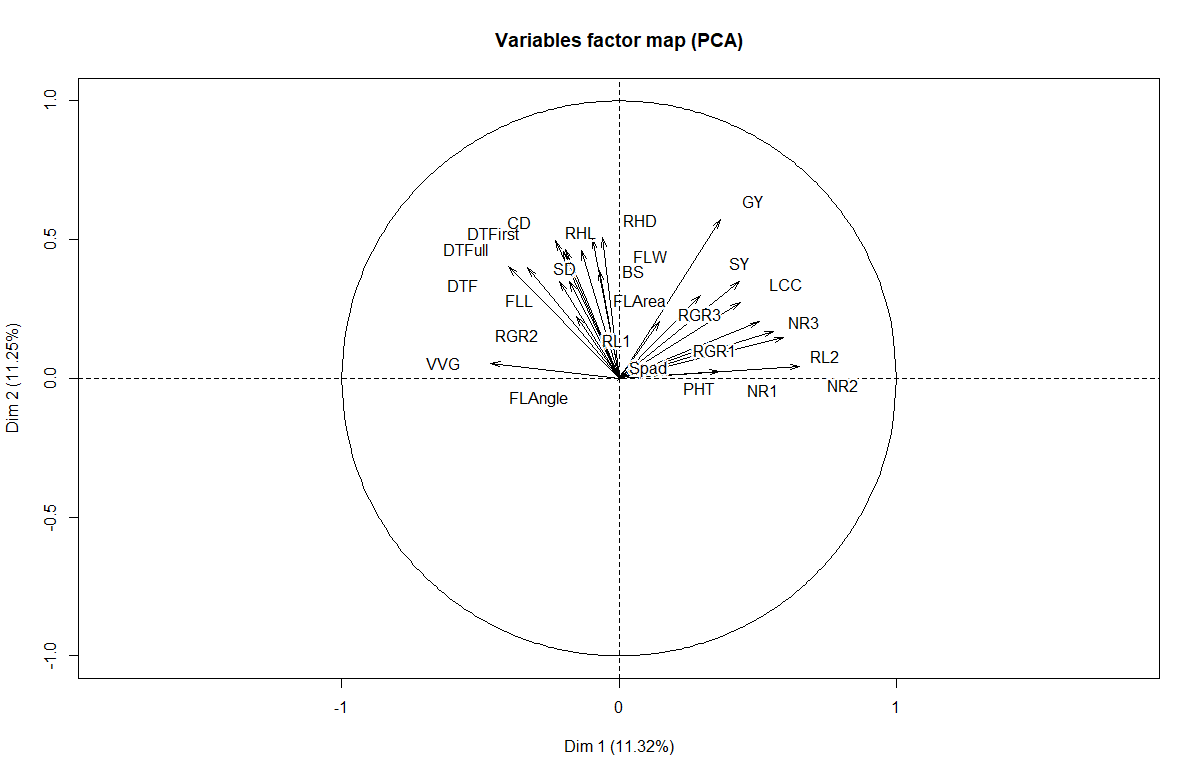
**

**(c)**

**Figure S4.** Principal component analysis plot of phenotypic traits in (a) 2015WS (b) 2016DS and (c) combined season
